# Arbuscular Mycorrhiza Augments Arsenic Tolerance in Wheat (*Triticum aestivum* L.) by Strengthening Antioxidant Defense System and Thiol Metabolism

**DOI:** 10.3389/fpls.2017.00906

**Published:** 2017-06-08

**Authors:** Surbhi Sharma, Garima Anand, Neeraja Singh, Rupam Kapoor

**Affiliations:** Department of Botany, University of DelhiNew Delhi, India

**Keywords:** arsenic, arbuscular mycorrhiza, oxidative stress, ascorbate-glutathione cycle, thiol metabolism, glyoxalase enzymes

## Abstract

Arbuscular mycorrhiza (AM) can help plants to tolerate arsenic (As) toxicity. However, plant responses are found to vary with the host plant and the AM fungal species. The present study compares the efficacy of two AM fungi *Rhizoglomus intraradices* (M1) and *Glomus etunicatum* (M2) in amelioration of As stress in wheat (*Triticum aestivum* L. var. HD-2967). Mycorrhizal (M) and non-mycorrhizal (NM) wheat plants were subjected to four levels of As (0, 25, 50, and 100 mg As kg^-1^ soil). Although As additions had variable effects on the percentage of root colonized by the two fungal inoculants, each mycobiont conferred benefits to the host plant. Mycorrhizal plants continued to display better growth than NM plants. Formation of AM helped the host plant to overcome As-induced P deficiency and maintained favorable P:As ratio. Inoculation of AMF had variable effects on the distribution of As in plant tissues. While As translocation factor decreased in low As (25 mg kg^-1^ soil), it increased under high As (50 and 100 mg As kg^-1^ soil). Further As translocation to grain was reduced (As grain:shoot ratio) in M plants compared with NM plants. Arsenic-induced oxidative stress (generation of H_2_O_2_ and lipid peroxidation) in plants reduced significantly by AMF inoculation. The alleviation potential of AM was more evident with increase in severity of As stress. Colonization of AMF resulted in higher activities of the antioxidant enzymes (superoxide dismutase, catalase, and guaiacol peroxidase). It increased the concentrations of the antioxidant molecules (carotenoids, proline, and α-tocopherol) than their NM counterparts at high As addition level. Comparatively higher activities of enzymes of glutathione-ascorbate cycle in M plants led to higher ascorbate:dehydroascorbate (AsA:DHA) and glutathione:glutathione disulphide (GSH:GSSG) ratios. Inoculation by AMF also augmented the glyoxalase system by increasing the activities of both glyoxalase I and glyoxalase II enzymes. Mycorrhizal colonization increased concentrations of cysteine, glutathione, non-protein thiols, and activity of glutathione-*S*-transferase that facilitated sequestration of As into non-toxic complexes. The study reveals multifarious role of AMF in alleviation of As toxicity.

## Introduction

Increase in geological and anthropogenic activities across the globe have led to heavy metal contamination in groundwater and soil. Heavy metals like As, cadmium (Cd), lead (Pb), chromium (Cr), and mercury (Hg) have been reported to be the major heavy metal pollutants of groundwater and soil (Chatterjee and Chatterjee, 2000; Öncel et al., 2000; Oancea et al., 2005). This has steered changes in physiological and biochemical processes in plants leading to a reduction in plant growth, performance and yield. Toxic effects of these heavy metals include inhibition of cytoplasmic enzymes and damage to cell structures due to oxidative stress (Van Assche and Clijsters, 1990; Asati et al., 2016). Groundwater contaminations with As have been reported in many countries such as Argentina, Bangladesh, Chile, China, India, Japan, Mexico, Mongolia, Nepal, Poland, Taiwan, Vietnam, and United States (Chowdhury et al., 2000; Smith et al., 2000; Anawar et al., 2002; Pandey et al., 2002). However, in the Ganges Delta region of Bangladesh and West Bengal, As in groundwater has emerged as the largest environmental health disaster (Rahman et al., 2009). Much of South Asia’s food grains supply particularly staple foods like wheat and rice comes from Indo-Gangetic Plain (IGP). Rice and wheat are the staple food crops occupying nearly 13.5 million hectares of the IGP of South Asia covering Pakistan, India, Bangladesh, and Nepal. These crops contribute more than 80% of the total cereal production and are critically important to employment and food security for hundreds of millions of rural families for these countries (Gupta and Seth, 2007; Sekar and Pal, 2012). As-contaminated groundwater is used for drinking as well as irrigation. There are concerns that As is absorbed by plants particularly cereals, that are irrigated with As-contaminated groundwater, and poses a great threat to human health and ecological safety (Zhu et al., 2008; Meharg et al., 2009).

The effect of the As-contaminated groundwater irrigation on crops has attracted attention only during the last decade (Norra et al., 2005; Huang et al., 2006; Bhattacharya et al., 2010). Most studies in the past have focused on rice, and relatively less information is available on As accumulation, distribution, and speciation in wheat, which is the second most important food grain cereal (Roychowdhury et al., 2002; Tao et al., 2006; Zhao et al., 2010; Tong et al., 2014).

Arsenic enters in the plants through phosphate transporters as a phosphate analog or through aquaglyceroporins (Sharma, 2012). The phytotoxic effects of As generally include reduction in growth, chlorophyll biosynthesis, and nutrient uptake (Moreno-Jiménez et al., 2012). Arsenate [As(V)] is the main As species occurring in aerobic soils. It acts as a phosphate (Pi) analog and is transported across the plasma membrane via phosphate transport systems (Stoeva and Bineva, 2003). Cytoplasmic As(V) interferes with metabolic processes involving Pi, making it potentially toxic to plants. However, it is rapidly reduced to arsenite (AsIII) in the cytoplasm (Meharg and Hartley-Whitaker, 2002; Stoeva and Bineva, 2003). Arsenate reduction results in the formation of ROS with consequent lipid peroxidation, and cellular damage (Meharg and Hartley-Whitaker, 2002; Sharma, 2012). Arsenite reacts with sulfhydryl groups (-SH) of enzymes and tissue proteins, inhibiting cellular function, causing death (Smith et al., 2010a). ROS produced as a result of As stress need to be scavenged for maintenance of normal plant growth. Detoxification mechanisms for As(III) include efflux from the roots, sequestration in cell vacuoles and complexation with thiols for which As(III) has very high-affinity (Smith et al., 2010a).

Earlier investigations have shown that higher plants that are adapted to As-polluted soils are generally associated with AM fungi (Meharg and Cairney, 1999; Gonzalez-Chavez et al., 2002). The mechanisms by which AM fungi augment plant tolerance to As stress are not clear. Potential mechanisms that have been frequently recognized are improved nutritional status and reduced metal uptake. Inoculation by AM fungi can exert protective effects on vascular plants under As contamination by transforming inorganic As in less toxic organic forms or by diluting As concentration by enhancing plant biomass (Gonzalez-Chavez et al., 2002; Liu et al., 2005; Chen et al., 2007; Dong et al., 2008; Zhang et al., 2015; Li et al., 2016).

Formation of AM provides an alternative pathway — AM pathway for uptake of nutrients especially P. The AM pathway is distinct from the direct pathway; it involves different cell types, different Pi transporters, and is likely to be separately regulated (Smith and Smith, 2011). The direct pathway involves high-affinity Pi transporters located in root hairs and epidermal cells near the root apex. However, the mycorrhizal pathway develops behind the root hair zone. It involves uptake of Pi by AM fungal high-affinity Pi transporters in the extraradical mycelium, followed by translocation of phosphorus along the hyphae to intracellular structures in the root cortex and transfer to the root. The transfer across the symbiotic interface involves efflux of Pi from the AM fungus into the apoplast and uptake into the plant cells by Pi transporter(s) that are preferentially or specifically expressed in colonized cortical cells. Mycorrhizal plants display lower specific As(V) uptake and higher P:As ratio than NM plants (Christophersen et al., 2009; Smith et al., 2010b).

The AM symbiosis is known to increase tolerance of plants to various abiotic stresses by promoting antioxidant defense system (Ruiz-Lozano, 2003; Wu et al., 2006; Evelin and Kapoor, 2014). However, information on antioxidant defense in mycorrhizal (M) versus non-mycorrhizal (NM) plants in relation to As stress is very scarce (Yu et al., 2009; Garg and Singla, 2012; Garg et al., 2015). Plants subjected to abiotic stress often produce toxic aldehydes such as methylglyoxal (MG). MG is mostly detoxified by the combined actions of the enzymes Gly I and Gly II that together with glutathione make up the glyoxalase system (Hoque et al., 2016). Several studies have shown close links between the antioxidant and glyoxalase systems in plants, suggesting a direct influence of the glyoxalase system on ROS detoxification (reviewed in Hoque et al., 2016). However, effect of AMF inoculation on glyoxalase system in relation to abiotic stress tolerance has not been reported so far.

Among the various detoxification pathways activated in the plants under As stress, increased synthesis of sulfur-containing defense compounds such as PCs and GSH is considered to be of prime importance for the tolerance and survival of plants (Mishra et al., 2009). Role of AM in modulating thiol metabolism in relation to heavy metal stress such as Cd and Zn has been recently realized (Garg and Kaur, 2013; Garg and Chandel, 2015). However, to the best of our knowledge there is no report on the effect of AM on thiol metabolism in plants under As-stress.

This study was aimed to compare the effectiveness of two AM fungal species (*Rhizoglomus intraradices* and *Glomus etunicatum*) on growth and tolerance of wheat (*Triticum aestivum* L. var. HD-2967) in different levels of As stress. We tested the hypotheses that (1) inoculation with AM fungi results in decreased As uptake and its translocation to shoot and grain; and (2) AM formation results in mitigation of As-induced oxidative stress. We also measured the concentration of molecules containing -SH groups (such as cysteine, glutathione, and NPSHs) to gain insights into broader aspects of AM mediated As-stress alleviation.

## Materials and Methods

### Plant Material

The crop plant chosen for the study is *T. aestivum* L. variety HD-2967. It is a double dwarf variety that was released for commercial cultivation in India in September 2011. It has profuse tillering and is resistant to Ug99, a deadly African race of stem rust already prevalent in central India. In the 2013–2014 Rabi (winter) season, it was grown in about six million hectares in India. The variety is resistant to salt stress, however, no information is available on tolerance to heavy metal stress including As.

### AMF Symbionts

Inocula for the AM fungal symbionts *R. intraradices* (N.C. Schenck and G.S. Sm.) C. Walker and A. Schüßler (CMCCWep319) and *G. etunicatum* W.N. Becker and Gerd (CMCC/AM-1207) were obtained from Center for Mycorrhiza Culture Collection, The Energy and Resources Institute, New Delhi, India. These were propagated as soil-based open cultures in sterile soil mixture (Kapoor et al., 2002). The cultures were maintained under natural conditions of temperature, light, and humidity for 1 year. No *Rhizobium* was added. NM mock inoculum consisted of trap plants without AMF inoculum grown under similar conditions. Before use, root colonization was confirmed and trap plants were allowed to dry. The inoculum consisted of the roots chopped into small pieces and mixed with the soil mass (containing about 150 spores per 10 g of soil and hyphae) of the culture pots.

### Soil and Arsenic Treatment

Physico-chemical properties of the soil used in this experiment were analyzed at Division of Soil Science and Agricultural Chemistry, Indian Agricultural Research Institute, New Delhi, India. The soil texture was sandy clay loam (48:29:23), pH 8.3 with high organic carbon concentration (1.0%), available phosphorus (116 kg ha^-1^) and potassium (432 kg ha^-1^) content. The soil used contained low nitrogen content (167 kg ha^-1^) but was adequate in micronutrients such as magnesium (97.3 mg kg^-1^), zinc (6.6 mg kg^-1^), and iron (9.4 mg kg^-1^). As content (14.8 μg kg^-1^) was also found in the soil. The soil had 11.5% moisture and 42.5% water holding capacity. Soil was mixed with sand in equal proportion. This soil mix will be referred to as soil henceforth. The soil was sterilized by autoclaving twice over 48 h at 121°C for 1 h, and 3 kg of sterilized soil was dispensed into each pot.

Different concentrations of As (0, 25, 50, and 100 mg As kg^-1^ soil) were prepared using sodium arsenate (Na_2_HAsO_4_.7H_2_O). These concentrations were chosen to study effectiveness of AM in low (25 mg As kg^-1^), moderate (50 mg As kg^-1^), and high (100 mg As kg^-1^) levels of As stress. Conclusion on these cardinal concentrations were based on studies on different crop plants (Chen et al., 2007; Liu et al., 2012; Spagnoletti and Lavado, 2015). In order to ensure homogenous distribution, As was dissolved in 50 ml of distilled water and then mixed thoroughly with soil. The pots were allowed to equilibrate for a period of 1 month by undergoing repeated cycles of saturation with distilled water and air drying (Cox and Kovar, 2001).

### Experimental Design

The experiment had a completely randomized factorial design with two factors: AMF inoculations and As levels. There were four levels of As (0, 25, 50, and 100 mg As kg^-1^, soil) as Na_2_HAsO_4_.7H_2_O and three AMF treatments [Control (NM), *R. intraradices*, and *G. etunicatum*] (**Supplementary Figure S1**). Hence, there were 12 treatments (4 × 3) and each treatment was replicated five times.

Wheat seeds were sterilized with 5% sodium hypochlorite solution for 15 min, and washed thoroughly with distilled water. The seeds were placed and allowed to germinate in wet sterilized germination paper for the period of 7 days at 25°C. Four seedlings (two-leaf stage) were transplanted in each pot. At the time of transplantation each seedling was inoculated by 20 g of AMF inoculum. NM plants were raised instead by adding dry mock inoculum.

The experiment was conducted in pots placed in the Botanical Garden of Department of Botany, University of Delhi, Delhi, India. Plants were grown for 45 days under natural conditions from December to February (Rabi season in India). During the experimental period, the average temperature ranged between 9 and 16°C and average relative humidity between 56 and 94%. Pots were casually rearranged after every 2 days during the growth period to take into account variations in environmental conditions (if any). The soil was maintained at 60% of field capacity, to avoid loss of solution due to drainage.

### Plant Harvest

Shoots and roots were harvested separately. Samples were carefully washed with deionized water to remove adhering soil particles. The dry weights of shoot and root were determined after oven drying at 70°C for 48 h. Wheat spikes were collected and dried at 70°C to constant weight. The spikes were then dehusked by hand and the weight of thousand grains was recorded.

### Percent Root Colonization by AMF

Subsamples of fresh roots were collected and cut into segments approximately 1 cm long, cleared with 10% (w/v) potassium hydroxide in a water bath at 90°C for 15 min, and stained with Trypan blue (Phillips and Hayman, 1970). The percentage of the total root colonized by the AM fungus was determined using the gridline intersect technique (Giovannetti and Mosse, 1980).

### Determination of As and P Concentrations

Roots, shoots, and grains were analyzed for concentration of As and P. Oven-dried plant tissue samples were powdered to pass through a 0.5-mm sieve. Samples were digested with nitric acid and hydrogen peroxide in kjeldahl tubes. After digestion the solutions were cooled, diluted to 50 ml using double distilled water, filtered into acid-washed plastic bottles. This solution was further used for the determination of As using atomic absorption spectrophotometer (AAS) (SensaAA, GBC, Hampshire, IL, United States). The solution of As acid (H_3_AsO_4_) in HNO_3_ (0.5 mol l^-1^ As Certipur^®^) was used as standard. The absorbance was read at 193.7 nm.

The concentration of P was determined using ammonium molybdate and stannous chloride (Allen, 1989). The absorbance was read at 700 nm.

### Measurement of Oxidative Damage

Lipid peroxidation in leaves and roots of wheat plants was detected according to Heath and Packer (1968) by measuring the concentration of MDA. The amount of MDA was calculated using extinction coefficient of 155 mM^-1^ cm^-1^ and expressed as nmol MDA g^-1^fresh weight.

Hydrogen peroxide concentrations in leaves and roots were determined according to Velikova et al. (2000). Plant tissue was homogenized in trichloroacetic acid in an ice bath. The supernatant obtained following centrifugation at 16099 × *g* for 15 min was used for determination of H_2_O_2_ concentration. The assay mixture contained supernatant, 10 mM phosphate buffer (pH 7.0), and 1 M potassium iodide (KI). The assay mixture was kept in dark for 1 h and the absorbance was read at 390 nm. The concentration of H_2_O_2_ was determined from a standard curve and expressed as H_2_O_2_ μg g^-1^ fresh weight.

### Preparation of Enzyme Extract

A fresh sample of leaf or root (1 g) was ground to a fine powder in liquid nitrogen and homogenized with a mortar and pestle in ice-cold 0.2 M phosphate buffer (pH 7.8) containing 0.1 mM EDTA. The homogenate was centrifuged at 16099 × *g* for 20 min at 4°C, and the supernatant was used as a crude enzyme source. For APX and DHAR activities, 1 mM AsA and 2 mM 2-mercaptoethanol were added into above phosphate buffer, respectively. The homogenate was centrifuged at 20,000 × *g* for 10 min at 4°C and supernatant was used as enzyme source.

An aliquot of the extract was used to determine protein concentration by the method of Bradford (1976) using bovine serum albumin as the standard. All enzyme activities were expressed as nkat mg^-1^ protein (1 katal = 1 mol s^-1^ catalytic activity) in all the treated as well as non-treated plants. All enzymatic measurements were carried out at 25 ± 2°C by using UV/Vis spectrophotometer (Beckman Coulter DU^®^730).

### Antioxidant Enzymes Activities

Superoxide dismutase (EC 1.15.1.1) activity was determined following the method by Elavarthi and Martin (2010). The reaction mix consisted of 50 mM phosphate buffer (pH 7.8), 2 mM EDTA, 9.9 mM L-methionine, 55 μM NBT, 0.025% triton X-100 and enzyme extract. Reaction was started by addition of 1 mM riboflavin followed by placing the test tubes under 20-W fluorescent bulbs for 15 min. A parallel set of tubes with same reaction mix was kept in dark that served as control, whereas those incubated in light without the enzyme extract served as blank. Activity was measured spectrophotometrically at 560 nm.

One unit of enzyme activity was defined as the amount of enzyme required to bring about 50% inhibition of the rate of NBT reduction measured at 560 nm. CAT (EC 1.11.1.6) activity was determined according to Aebi and Lester (1984) by monitoring the decrease in absorbance at 240 nm due to decomposition of H_2_O_2_. GPX (EC 1.11.1.7) activity was assayed as the increase in optical density at 470 nm due to the oxidation of guaiacol to tetra-guaiacol (Egley et al., 1983).

### Non-enzymatic Antioxidants

Carotenoids in leaves were extracted in dimethyl sulfoxide and their concentration (mg g^-1^ fresh weight) was calculated using the formula given by Arnon (1949). For proline estimation, plant tissue was homogenized in 3% sulfosalicylic acid following the method of Bates et al. (1973). The reaction mixture consisted of acid ninhydrin reagent, glacial acetic acid and supernatant. Toluene was added to the reaction mixture, and absorbance was recorded at 520 nm. The concentration of α-tocopherol (μg g^-1^ fresh weight) was determined according to Sadasivam and Manickam (2008).

### Components of Ascorbate-Glutathione Cycle

Ascorbate peroxidase (EC 1.11.1.11) activity was estimated following the protocol of Nakano and Asada (1981). The enzyme activity was calculated by using an extinction coefficient of 2.8 mM^-1^cm^-1^. GR (EC 1.6.4.2) activity was measured according to Smith et al. (1989) by monitoring the reduction of 5-5′-dithiobis(2-nitrobenzoic acid) (DTNB) to 5-thionitrobenzoic acid (TNB) by glutathione in the reaction at 412 nm.

Monodehydroascorbate reductase (EC 1.6.5.4) activity was assessed according to the method of Miyake and Asada (1992). The decrease in absorbance was read at 340 nm.

Dehydroascorbate reductase (EC 1.8.5.1) activity was assayed by following the method of Nakano and Asada (1981). An increase in absorbance was read at 265 nm, and DHAR activity was calculated using an extinction coefficient of 7.0 mM^-1^ cm^-1^. One unit (U) of enzyme activity is defined as 1 nmol DHA reduced min^-1^.

Concentrations of ascorbate (AsA) and dehydroascorbate (DHA) were determined using the methods of Arakawa et al. (1981) and Nakagawara and Sagisaka (1984). 0.1 g of fresh tissue (roots and leaves) was homogenized in 10 ml of cold 5% (w/v) trichloroacetic acid. The homogenate was centrifuged at 16,000 × *g* for 10 min at 4°C and the supernatant was used for the AsA and total ascorbate (AsA + DHA) assay. Total ascorbate was determined through a reduction of DHA to ascorbate by dithiothreitol (DTT). DHA concentrations were estimated subtracting AsA from total ascorbate. A standard curve in the range 0–10 μmol of ascorbate or DHA was plotted to find out the related contents.

Total glutathione was determined by following the method of Wang and Jiao (2000). Estimation of GSH was done following the method of Wu et al. (2006). GSSG was obtained by subtracting GSH from total GSH.

### Enzymes of Glyoxalase System

Glyoxalase I (EC 4.4.1.5) activity was determined according to the method of Hossain et al. (2009). Briefly, the assay mixture contained 100 mM K-phosphate buffer (pH 7.0), 15 mM magnesium sulfate, 1.7 mM GSH, and 3.5 mM MG in a final volume of 700 μl. The increase in absorbance was recorded at 240 nm for 1 min and the activity was calculated using the extinction coefficient of 3.37 mM^-1^ cm^-1^.

Glyoxalase II (EC 3.1.2.6) activity was assessed following the method of Principato et al. (1987) by examining the formation of GSH at 412 nm for 1 min. The increase in absorbance was recorded at 412 nm for 1 min and the activity was computed using the extinction coefficient of 13.6 mM^-1^ cm^-1^.

### Thiol Metabolites and Related Enzyme

Cysteine concentration was estimated according to Gaitonde (1967). The reaction mixture consisted of acid ninhydrin reagent II, glacial acetic acid and supernatant. Following the development of pink color absorbance was recorded at 560 nm. Cysteine concentration was calculated using 28 mM^-1^ cm^-1^ as extinction coefficient, expressed as nmol g^-1^ fresh weight. NPSHs were determined following Ellman (1959) using Ellman’s reagent (5 mM EDTA, 0.6 mM DTNB present in 0.12 M phosphate buffer, pH 7.5). Concentration of NPSHs was calculated using 13.1 mM^-1^ cm^-1^ as extinction coefficient and expressed as nmol g^-1^ fresh weight. PCs were determined by calculating the difference between total NPSHs and GSH (Bhargava et al., 2005).

Glutathione-*S*-transferase (EC 2.5.1.18) activity was estimated by the method of Habig and Jakoby (1981).

### Statistical Analysis

All the numerical data obtained from the experiments was analyzed using Statistical Package for the Social Sciences 21.0 (SPSS Inc., Armonk, NY, United States; IBM Corporation, United States). One-way ANOVA was performed for comparing significant differences among individual means. Two-way ANOVA was performed for studying interaction between mycorrhizal and As treatments. Differences between the individual means were compared using Duncan’s test.

## Results

**FIGURE 1:**
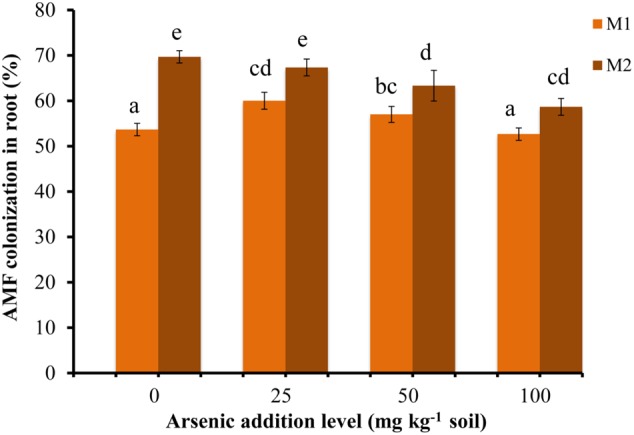
Effect of As addition levels on *Triticum aestivum* L. var. HD-2967 root colonization by *Rhizoglomus intraradices* (M1) and *Glomus etunicatum* (M2). Data is represented as mean ± SD (*n* = 5). Bars showing different letters indicate significant differences among treatments according to the Duncan’s multiple comparison test (*p* < 0.05); where M1, *R. intraradices*; M2, *G. etunicatum.*

*Triticum aestivum* var. HD2967 plants successfully formed AM when inoculated with *R. intraradices* (M1) or *G. etunicatum* (M2) (**Supplementary Figure S2**). The percent root colonization ranged between 52 and 61% for M1 and between 58 and 70% for M2. Mock-inoculated plants stayed NM (**Figure 1**). The percent root colonization in M2 fungal species decreased with increase in As in soil. While it increased in M1 plants at lower addition levels of As (25 mg kg^-1^ soil) and then gradually decreased at higher concentrations (50 and 100 mg kg^-1^ soil). Mycorrhizal colonization was significantly influenced by As, AMF and their interaction (**Table 1**).

**Table 1:** Two-way ANOVA analysis of Arsenic addition levels (As), mycorrhizal treatments (AMF) and their interactions (As × AMF) on variables of *Triticum aestivum* L. var. HD-2967 studied.

Significance	SHOOT			ROOT		
	As	AMF treatments	As × AMF	As	AMF treatments	As × AMF
Dry weight	^∗∗∗^	^∗∗∗^	^∗∗∗^	^∗∗∗^	^∗∗∗^	^∗∗∗^
Thousand grain weight	^∗∗∗^	^∗∗∗^	^∗^	nd	nd	nd
As concentration	^∗∗∗^	^∗∗∗^	ns	^∗∗∗^	^∗∗∗^	ns
P concentration	^∗∗∗^	^∗∗∗^	^∗∗∗^	^∗∗∗^	^∗∗∗^	^∗∗∗^
H_2_O_2_ concentration	^∗∗∗^	^∗∗∗^	^∗∗∗^	^∗∗∗^	^∗∗∗^	^∗∗∗^
MDA concentration	^∗^	^∗∗∗^	ns	^∗∗∗^	^∗∗∗^	^∗∗∗^
SOD activity	^∗∗∗^	^∗∗∗^	^∗∗^	^∗∗∗^	^∗∗∗^	^∗∗∗^
CAT activity	^∗∗∗^	^∗∗∗^	^∗∗∗^	^∗∗∗^	^∗∗∗^	^∗∗∗^
GPX activity	^∗∗∗^	^∗∗∗^	^∗∗∗^	^∗∗∗^	^∗∗∗^	^∗∗∗^
APX activity	^∗∗∗^	^∗∗∗^	^∗∗^	^∗∗∗^	^∗∗∗^	^∗∗∗^
Carotenoids concentration	^∗∗∗^	^∗∗∗^	^∗∗∗^	nd	nd	nd
Proline concentration	^∗∗∗^	^∗∗∗^	^∗∗∗^	^∗∗∗^	^∗∗∗^	^∗∗∗^
α**-**Tocopherol concentration	^∗∗∗^	^∗∗∗^	^∗∗∗^	^∗∗∗^	^∗∗∗^	^∗∗∗^
Total ascorbate concentration	^∗∗∗^	^∗∗∗^	^∗∗∗^	^∗∗∗^	^∗∗∗^	^∗∗∗^
MDHAR activity	^∗∗^	^∗∗∗^	^∗∗^	ns	^∗∗∗^	^∗∗∗^
DHAR activity	^∗∗∗^	^∗∗∗^	^∗∗∗^	^∗∗∗^	^∗∗∗^	^∗∗∗^
GSH concentration	^∗∗∗^	^∗∗∗^	^∗∗∗^	^∗∗∗^	^∗∗∗^	^∗∗∗^
GSSG concentration	^∗∗∗^	^∗∗∗^	^∗∗∗^	^∗∗∗^	^∗∗∗^	^∗∗∗^
GR activity	^∗∗∗^	^∗∗∗^	^∗∗∗^	^∗∗∗^	^∗∗∗^	^∗∗∗^
Gly I activity	^∗∗∗^	^∗∗∗^	^∗∗∗^	^∗∗∗^	^∗∗∗^	^∗^
Gly II activity	^∗∗∗^	^∗∗∗^	^∗^	^∗∗∗^	^∗∗^	ns
Cysteine concentration	^∗∗∗^	^∗∗∗^	^∗∗∗^	^∗∗∗^	^∗∗∗^	^∗∗∗^
NPSHs concentration	^∗∗∗^	^∗∗∗^	^∗∗^	^∗∗∗^	^∗∗∗^	^∗∗∗^
PC concentration	^∗∗∗^	^∗∗∗^	^∗∗∗^	^∗∗∗^	^∗∗∗^	^∗∗∗^
GST activity	^∗∗∗^	^∗∗∗^	^∗∗∗^	^∗∗∗^	^∗∗∗^	^∗∗∗^

*Where nd, not determined; ns, not significant; *^∗∗∗^ p* < 0.001, ^∗∗^*p* < 0.01, ^∗^*p* < 0.05.*

Two-way analysis revealed significant influence of As and AMF inoculation on dry weights of shoots as well as roots (**Table 1**). In response to increase in As concentrations, wheat plants exhibited decrease in plant biomass (**Table 2**). The reduction was seen in both NM and M plants and was dependent on As concentration in soil. However, at all concentrations of As, M plants showed higher biomass as compared to NM plants with an exception of shoot parameters at 50 mg As kg^-1^ soil for M1. Response to mycorrhizal treatment was most evident at 100 mg As kg^-1^ soil, where shoot and root biomass of M wheat was near twofold (M1) or more than twofolds (M2) that of NM. Overall the performance of wheat plants with respect to its biomass was highest in M2. Biomass of grains (1000 grains) decreased with increase in As stress, with grains from NM plants registering a greater decline than M plants. At 100 mg As kg^-1^ soil treatment, grain biomass declined in NM by 44% while in M1 and M2 it declined by 36% over their respective controls (0 mg As kg^-1^ soil).

**Table 2:** Effect of As addition levels and AMF treatments on biomass of various parts of *T. aestivum* L. var. HD-2967 plants.

As addition levels (mg kg^-1^ soil)	AMF treatment	Dry weight (g)		
		Shoot	Root	Grain (1000)
0	NM	3.0 ± 0.07 f	1.5 ± 0.02 e	43.7 ± 1.01 e
	M1	3.9 ± 0.09 g	1.9 ± 0.02 h	52.7 ± 1.54 f
	M2	4.1 ± 0.05 g	1.9 ± 0.04 h	55.2 ± 1.18 f
25	NM	1.9 ± 0.02 d	1.1 ± 0.02 c	37.6 ± 0.95 d
	M1	2.2 ± 0.24 e	1.6 ± 0.03 f	42.1 ± 0.10 e
	M2	3.9 ± 0.04 g	1.8 ± 0.04 g	53.8 ± 0.85 f
50	NM	1.0 ± 0.02 b	0.9 ± 0.03 b	29.9 ± 1.01 b
	M1	0.9 ± 0.03 ab	1.4 ± 0.02 d	35.3 ± 1.08 cd
	M2	2.4 ± 0.15 e	1.7 ± 0.03 f	42.7 ± 1.11 e
100	NM	0.7 ± 0.21 a	0.6 ± 0.01 a	24.6 ± 1.26 a
	M1	1.4 ± 0.04 c	1.1 ± 0.01 c	33.9 ± 4.62 c
	M2	1.8 ± 0.03 d	1.4 ± 0.02 d	35.3 ± 1.45 cd

*Data is presented as mean ± SD (*n* = 5). Different letters indicate significant differences among treatments according to the Duncan’s multiple comparison test (*p* < 0.05); where NM, non-mycorrhizal, M1, *Rhizoglomus intraradices*; M2, Glomus etunicatum.*

### As in Plant Tissue

The concentration of As increased steadily in shoots and roots of both NM and M plants with increasing amount of As in soil (**Table 3**). Two-way ANOVA analysis showed significant independent effects of As and AMF on concentration of As in root and shoot, however, no significant As × AMF interaction was observed (**Table 1**). The inoculation with M1 or M2 significantly reduced As concentration in both shoots and roots with respect to NM (**Table 3**). As concentration decreased in shoots of M1 plants by 52, 5.83, and 7.20%, and that of M2 plants by 54, 14.8, and 10.1% at 25, 50, and 100 mg As kg^-1^ soil, respectively, when compared with NM counterparts. In roots, inoculation with M1 decreased As concentration by 21.7, 11.2, and 18.5% over NM plants at 25, 50, and 100 mg As kg^-1^ soil, respectively. Whereas M2 decreased As concentration by 36.3, 36.5, and 29.7% at 25, 50, and 100 mg As kg^-1^soil as compared to NM. Thus the proportion of decrease was higher in roots compared to shoots in M plants at higher concentrations. However, in contrast the content of As in M plant tissue (shoot and root) was higher than NM plants at higher levels of As stress. As content was maximum in tissues of M2 plants followed by M1 and NM at 100 mg As kg^-1^ soil.

**Table 3:** Effect of As addition levels and AMF treatments on As concentration, As content, and phosphorus concentration in *T. aestivum* L. var. HD-2967 plants.

As addition levels (mg kg^-1^ soil)	AMF treatment	As concentration (μg g^-1^ dry weight)			Arsenic content (g)		P concentration (mg g^-1^)	
		Shoot	Root	Grain	Shoot	Root	Shoot	Root
0	NM	ND	ND	ND	ND	ND	6.9 ± 0.06 g	6.2 ± 0.07 gh
	M1	ND	ND	ND	ND	ND	7.2 ± 0.04 h	6.7 ± 0.14 i
	M2	ND	ND	ND	ND	ND	7.8 ± 0.13 k	6.7 ± 0.12 i
25	NM	11.1 ± 0.7 b	15.4 ± 0.6 b	0.8 ± 0.06 d	21.7 ± 1.9 c	17.4 ± 0.7 a	6.5 ± 0.03 f	5.5 ± 0.03 f
	M1	5.3 ± 0.3 a	12.0 ± 0.5 a	0.5 ± 0.02 c	11.9 ± 2.2 b	19.4 ± 1.3 a	7.4 ± 0.02 i	6.0 ± 0.08 g
	M2	5.1 ± 0.1 a	9.8 ± 0.2 a	0.3 ± 0.01 a	19.7 ± 0.4 bc	17.7 ± 0.7 a	7.5 ± 0.04 j	6.3 ± 0.14 h
50	NM	22.8 ± 1.3 d	39.9 ± 3.5 e	0.8 ± 0.05 d	21.7 ± 2.1 c	37.5 ± 3.3 b	5.5 ± 0.04 d	4.1 ± 0.33 c
	M1	21.5 ± 0.9 d	35.4 ± 2.5 d	0.6 ± 0.04 c	20.8 ± 0.8 bc	48.8 ± 4.5 d	5.6 ± 0.02 d	4.4 ± 0.14 d
	M2	19.4 ± 0.1 c	25.3 ± 1.5 c	0.5 ± 0.02 b	46.0 ± 3.6 de	41.8 ± 2.5 c	6.0 ± 0.08 e	5.1 ± 0.02 e
100	NM	31.5 ± 0.3 f	53.8 ± 1.7 g	2.0 ± 0.04 g	22.4 ± 7.9 c	33.7 ± 2.2 b	3.5 ± 0.01 a	2.9 ± 0.04 a
	M1	29.2 ± 2.7 e	43.8 ± 1.7 f	1.5 ± 0.04 f	39.5 ± 3.4 d	48.4 ± 2.9 d	4.7 ± 0.02 b	3.3 ± 0.04 b
	M2	28.3 ± 0.7 e	37.8 ± 2.8 de	1.1 ± 0.08 e	52.3 ± 2.5 e	51.2 ± 4.8 d	5.1 ± 0.05 c	3.9 ± 0.03 c

*Data is presented as mean ± SD (*n* = 5). Different letters indicate significant differences among treatments according to the Duncan’s multiple comparison test (*p* < 0.05); where NM, non-mycorrhizal; M1, *R. intraradices*; M2, *G. etunicatum*; ND, not detected.*

Arsenic concentration in grain increased with increase in As in soil (**Table 3**). However, at all levels of As in soil, its concentration was far less in grains of M plants compared to NM plants. Arsenic concentration declined by 28, 27, and 24.5% in M1 and by 60, 42, and 45% in M2 at 25, 50, and 100 mg As kg^-1^soil compared to their NM counterparts.

### P Concentration

Mycorrhizal condition, level of As in soil and their interaction had strong effects on P concentration in shoot and root of wheat plants (**Table 1**). P concentration markedly declined in plants in response to As exposure (**Table 3**). Inoculation by AMF alleviated the antagonistic effect of As, and significantly enhanced P-accumulation in both shoot and root compared to NM counterparts at all levels of As. At 100 mg As kg^-1^ soil, P-uptake was most adversely affected, and the ameliorative effect of AMF was also evident at this As level. At all levels of As in soil, P:As ratio was higher in M over NM plants (**Table 4**).

**Table 4:** Effect of As addition levels and AMF treatments on phosphorus:arsenic ratio, As translocation from root to shoot and shoot to grain and bioaccumulation factor in *T. aestivum* L. var. HD-2967 plants.

As addition levels (mg kg^-1^ soil)	AMF treatment	Phosphorus:Arsenic ratio		Translocation factor		Bioaccumulation factor
		Shoot	Root	Shoot:Root	Grain:Shoot	
0	NM	ND	ND	ND	ND	ND
	M1	ND	ND	ND	ND	ND
	M2	ND	ND	ND	ND	ND
25	NM	582.6	354.8	0.7	0.06	0.6
	M1	1384.6	502.5	0.4	0.10	0.5
	M2	1462.9	647.6	0.5	0.05	0.4
50	NM	241.3	102.3	0.5	0.03	0.8
	M1	261.4	123.2	0.6	0.02	0.7
	M2	308.6	202.2	0.8	0.02	0.5
100	NM	111.7	54.2	0.6	0.06	0.5
	M1	161.0	76.0	0.7	0.05	0.4
	M2	180.7	103.6	0.7	0.03	0.4

*NM, non-mycorrhizal; M1, *R. intraradices*; M2, *G. etunicatum*; ND, not detected.*

### Translocation and Bioaccumulation Factor

The TF decreased in NM plants with increase in concentration of As in soil (**Table 4**). Inoculation by AMF showed variable effect on TF (shoot:root), while in low dose of As (25 mg kg^-1^soil), there was decline in TF in M plants. In moderate and high levels of As, in contrast to decrease in NM, significant increase in TF was observed in M1 and M2 treatments. However, translocation of As from shoot to grains was lesser in M plants (M1 and M2) than NM plants (**Table 4**). The As bioaccumulation factor decreased with increase up to 50 mg As kg^-1^ soil, and further decreased with higher concentration in all the treatments. Mycorrhizal plants accumulated less amount of As as compared to NM plants with M2 plants showing a less As accumulation than M1 plants.

### Oxidative Damage

**FIGURE 2:**
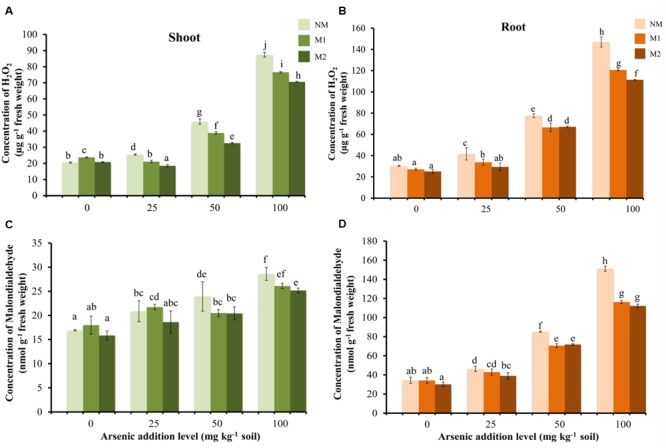
Effect of As addition levels and AMF treatments on concentrations of H_2_O_2_ in **(A)** shoot and **(B)** root and MDA in **(C)** shoot and **(D)** root of *T. aestivum* L. var. HD-2967 plants. Data is represented as mean ± SD (*n* = 5). Bars showing different letters indicate significant differences among treatments according to the Duncan’s multiple comparison test (*p* < 0.05); where NM, non-mycorrhizal; M1, *R. intraradices*; M2, *G. etunicatum.*

With increase in As in soil, there was a gradual increase in H_2_O_2_ concentrations in M and NM wheat plants (**Figures 2A,B**). The H_2_O_2_ level was higher in roots as compared to shoots. In shoots, the H_2_O_2_ concentration was significantly influenced by As stress, AMF treatments and their interaction (**Table 1**). M2 plants continued to maintain lowest H_2_O_2_ concentration level followed by M1 and NM plants. Regardless of the intensity of As toxicity, M plants displayed lower H_2_O_2_ concentration than corresponding NM plants; however, the differences were not significant between AMF treatments in roots up to 50 mg As kg^-1^soil (**Figure 2B**).

Lipid peroxidation (measured as concentration of MDA) showed a linear increase with corresponding increase in the concentration of As in the soil (**Figures 2C,D**). Again the level of lipid peroxidation was very high in root in comparison to shoot in all the treatments. Mycorrhizal plants showed lower levels of lipid peroxidation than the corresponding NM plants at each As level (except M1 at 25 mg As kg^-1^ soil in shoot). Two-way analysis revealed significant interaction between As concentration and AMF inoculation on root MDA concentration whereas no significant effect of their interaction was observed in shoot MDA concentrations (**Table 1**).

### Antioxidant Enzyme Activities

**FIGURE 3:**
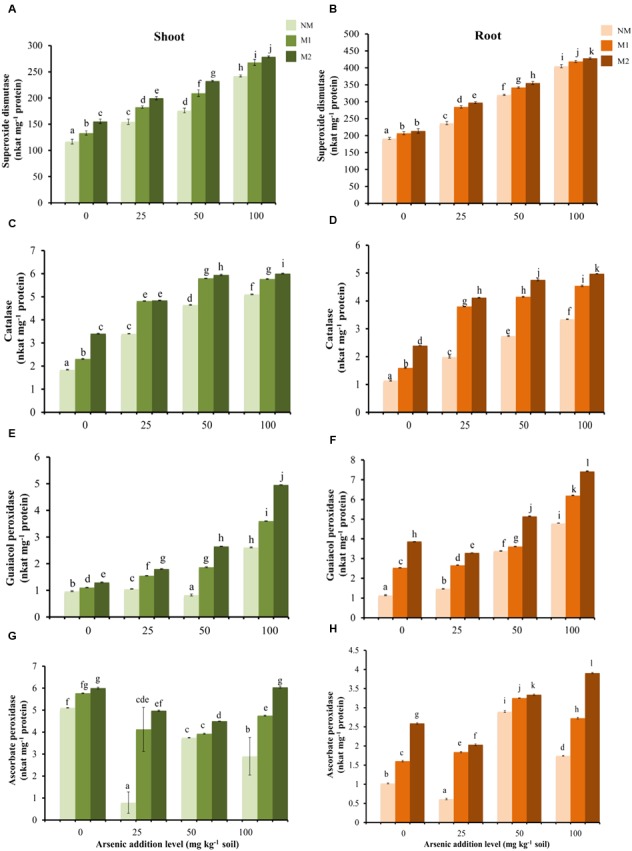
Effect of As addition levels and AMF treatments on the activities of SOD in **(A)** shoot and **(B)** root, CAT in **(C)** shoot and **(D)** root, GPX in **(E)** shoot and **(F)** root and APX in **(G)** shoot and **(H)** root of *T. aestivum* L. var. HD-2967 plants. Data is represented as mean ± SD (*n* = 5). Bars showing different letters indicate significant differences among treatments according to the Duncan’s multiple comparison test (*p* < 0.05); where NM, non-mycorrhizal; M1, *R. intraradices*; M2, *G. etunicatum*.

Two-way ANOVA showed significant influence of As, AMF and interactive effects of As × AMF on activities of SOD, CAT, GPX, and APX in shoots as well as roots (**Table 1**). SOD activity was highest among all the antioxidant enzyme activities (**Figures 3A,B**). The increase in As-induced SOD activity led to concomitant increment in activities of H_2_O_2_ scavenging enzymes — CAT and GPX (SOD-CAT: *r*_shoot_ = 0.91; *r*_root_ = 0.79; SOD-GPX: *r*_shoot_ = 0.90; *r*_root_ = 0.86). The activities of these enzymes were higher in tissues of M plants (**Figures 3C–F**). The APX activity increased in plants with increase in intensity of As stress from 25 to 50 mg As kg^-1^ soil, thereafter it decreased at 100 mg As kg^-1^ soil in roots of NM and M1 plants. The enzyme activity was higher in M plants (**Figures 3G,H**).

### Non-enzymatic Antioxidants Molecules

Two-way ANOVA showed significant influence of As, AMF individually and their interactive effects on carotenoids, proline, and α-tocopherol concentrations (**Table 1**). The concentration of carotenoids increased at initial levels of As concentration (25 mg As kg^-1^ soil) in NM and M plants. The concentration declined with increase in As at 50 and 100 mg As kg^-1^ soil, but the concentration of carotenoids in M plants (M1 and M2) at 50 mg As kg^-1^ soil was higher than the concentrations at 0 mg As kg^-1^ soil, similarly in M2 plants the carotenoid concentration increased at 100 mg As kg^-1^ soil (**Table 5**). Inoculation of AMF led to an overall increased level of carotenoids as compared to the NM plants, although this extent of increase varied with each As addition level.

**Table 5:** Effect of As addition levels and AMF treatments on concentrations of carotenoids, proline, α-tocopherol and total ascorbate in *T. aestivum* L. var. HD-2967 plants.

As addition levels (mg kg^-1^ soil)	AMF treatment	Carotenoids(μg g^-1^ fresh weight)	Proline(μg g^-1^ fresh weight)		α-Tocopherol(μg g^-1^ fresh weight)		Total ascorbate(μg g^-1^ fresh weight)	
		Shoot	Shoot	Root	Shoot	Root	Shoot	Root
0	NM	766.7 ± 13.7 b	57.2 ± 1.8 a	78.7 ± 0.9 a	7.52 ± 0.9 a	4.9 ± 1.2 a	125.5 ± 4.9 a	268.8 ± 7.7 a
	M1	970.0 ± 8.9 e	99.5 ± 0.5 b	188.7 ± 2.7 b	9.92 ± 0.8 a	7.4 ± 1.4 ab	235.5 ± 5.0 c	300.0 ± 3.4 b
	M2	900.0 ± 8.9 d	127.9 ± 2.4 c	287.0 ± 3.2 c	9.55 ± 0.5 a	7.9 ± 0.9 b	184.2 ± 10.5 b	382.6 ± 6.5 d
25	NM	863.3 ± 18.6 c	224.3 ± 2.8 c	275.33 ± 4.3 d	9.3 ± 0.7 a	7.4 ± 1.4 b	176.1 ± 1.9 b	328.8 ± 5.2 c
	M1	1060.0 ± 8.9 g	81.3 ± 1.1 d	124.19 ± 2.7 e	19.9 ± 1.7 c	16.0 ± 0.7 c	259.9 ± 4.3 cd	481.0 ± 4.9 f
	M2	1110.0 ± 17.9 h	122.7 ± 1.7 e	236.40 ± 5.6 g	24.9 ± 1.1 d	20.8 ± 0.7 d	279.3 ± 6.8 d	482.1 ± 7.0 f
50	NM	763.3 ± 5.2 b	150.6 ± 17.5 d	322.18 ± 3.0 g	9.8 ± 2.0 a	9.1 ± 0.4 b	382.6 ± 6.5 e	426.6 ± 5.8 e
	M1	1016.7 ± 27.3 f	247.7 ± 1.0 e	407.04 ± 5.5 h	21.8 ± 1.2 cd	16.7 ± 0.4 c	432.9 ± 11.0 f	512.3 ± 2.7 g
	M2	966.7 ± 5.2 e	99.4 ± 0.4 f	156.56 ± 0.9 i	45.9 ± 1.2 f	23.3 ± 0.9 de	550.6 ± 16.3 g	517.2 ± 7.5 g
100	NM	576.7 ± 5.2 a	148.5 ± 1.6 g	281.53 ± 0.5 f	13.7 ± 1.0 b	9.1 ± 0.5 b	403.3 ± 8.0 e	427.4 ± 6.4 e
	M1	980.0 ± 8.9 e	199.6 ± 0.3 h	350.91 ± 1.6 j	36.4 ± 1.2 e	25.1 ± 0.5 e	658.5 ± 8.5 h	760.2 ± 5.7 h
	M2	1126.7 ± 13.6 h	273.8 ± 0.8 i	441.11 ± 2.5 k	48.8 ± 1.5 f	35.1 ± 0.6 f	751.2 ± 14.0 i	936.3 ± 5.9 i

*Data is presented as mean ± SD (*n* = 5). Different letters indicate significant differences among treatments according to the Duncan’s multiple comparison test (*p* < 0.05); where NM, non-mycorrhizal; M1, *R. intraradices*; M2, G. etunicatum.*

Proline concentration increased at lower addition levels of As (25 mg As kg^-1^ soil) in NM plants while it decreased in M plants in both shoot and root. As the addition levels of As increased (50 mg As kg^-1^soil) proline concentration in shoots decreased in NM and M2 plants, showing a hike in M1 plants. In roots, the concentration increased in M1 and NM plants while it decreased in M2 plants. With a higher addition level of As (100 mg As kg^-1^soil) proline concentration decreased in both shoot and root of NM and M1 plants and increased in M2 plants. Both the mycobionts did not show a definite pattern of increase reporting that both AMF responded differently to each As treatment. However, at highest As addition level (100 mg As kg^-1^soil) proline concentration was higher in M plants than NM plants both in shoot and root. Concentration of α-tocopherol enhanced on increasing the As stress in NM, M1, and M2 plants; however, in plants inoculated with M1 and M2, level of α-tocopherol was higher than that of NM plants.

### Components of Ascorbate-Glutathione Cycle

**FIGURE 4:**
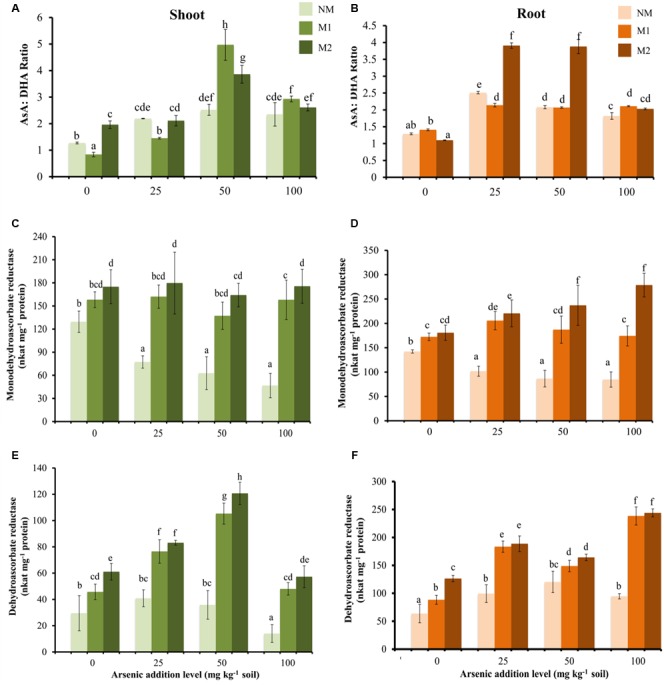
Effect of As addition levels and AMF treatments on the ratio of AsA:DHA in **(A)** shoot and **(B)** root, activities of MDHAR in **(C)** shoot and **(D)** root and DHAR in **(E)** shoot and **(F)** root of *T. aestivum* L. var. HD-2967 plants. Data is represented as mean ± SD (*n* = 5). Bars showing different letters indicate significant differences among treatments according to the Duncan’s multiple comparison test (*p* < 0.05); where NM, non-mycorrhizal; M1, *R. intraradices*; M2, *G. etunicatum.*

The concentrations of AsA, DHA were influenced by As treatments, consequently AsA:DHA ratio also changed (**Figures 4A,B**). In roots the value was highest in M2 plants at 25 and 50 mg As kg^-1^soil, while slight effect of AMF treatment was evident at 100 mg As kg^-1^ soil. The total ascorbate concentration significantly increased with increase up to 50 mg As kg^-1^ soil, beyond this the change was not significant in NM plants (**Table 5**). However, M plants sustained increase in total ascorbate with increase in As stress

At all levels of As stress, MDHAR activity was higher in M plants as compared with that of NM plants (**Figures 4C,D**). Similarly, at all levels of As, DHAR activity was higher in M plants (**Figures 4E,F**). Interestingly, in shoot DHAR showed strong positive correlation with GSH and GSSG (DHAR-GSH: *r*_shoot_ = 0.83; DHAR-GSSG: *r*_shoot_ = 0.84), in contrast in root it showed weak negative correlation with these parameters (DHAR-GSH: *r*_root_ = -0.33; DHAR-GSSG: *r*_root_ = -0.44).

**FIGURE 5:**
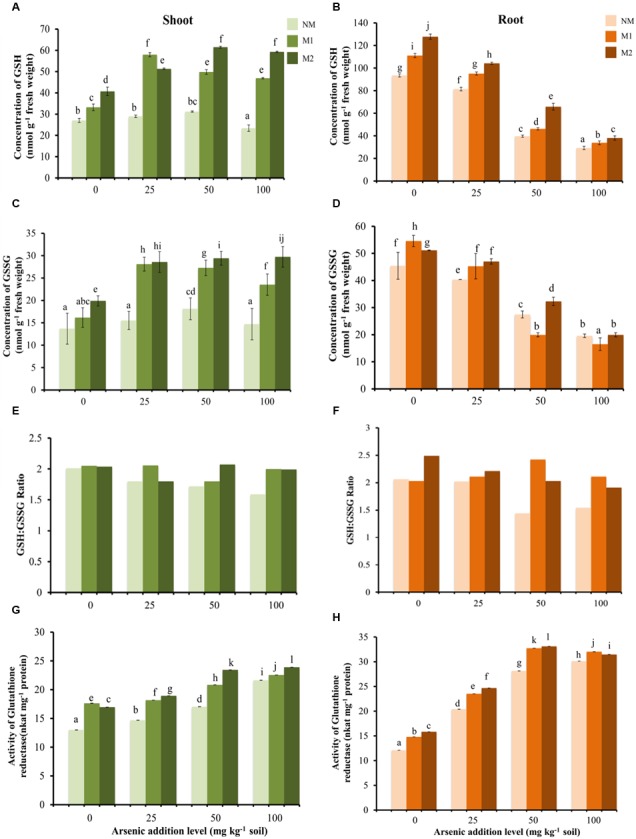
Effect of As addition levels and AMF treatments on concentrations of GSH in **(A)** shoot and **(B)** root, GSSG in **(C)** shoot and **(D)** root, ratio of GSH:GSSG in **(E)** shoot and **(F)** root and the activity of GR in **(G)** shoot and **(H)** root of *T. aestivum* L. var. HD-2967 plants. Data is represented as mean ± SD (*n* = 5). Bars showing different letters indicate significant differences among treatments according to the Duncan’s multiple comparison test (*p* < 0.05); where NM, non-mycorrhizal; M1, *R. intraradices*; M2, *G. etunicatum.*

Two-way ANOVA revealed significant influence of As, AMF and interactive effects of As × AMF on concentration of GSH in shoot as well as root (**Table 1**). In shoot, As exposure to plants resulted in varied response in different AMF treatments in terms of GSH concentration (**Figure 5A**). With increase in As in soil from 0 to 50 mg As kg^-1^soil, a significant increase in concentration of GSH was observed, with an exception of M1 plants where GSH concentration showed a striking increase at initial levels of As (25 mg kg^-1^ soil) but thereafter declined at 50 mg As kg^-1^ soil. With further escalation in As to 100 mg kg^-1^ soil, a decrease in GSH level was observed in NM plants while M1 and M2 plants showed non-significant change. In root, there was a steady decline in concentration of GSH with increasing concentration of As (**Figure 5B**). However, in M plants the GSH concentration was greater as compared to NM plants.

Two-way ANOVA revealed significant influence of As, AMF and interactive effects of As × AMF on the concentration of GSSG in shoot as well as root (**Table 1**). In shoot, at all levels of As stress the concentration of GSSG was highest in M2 followed by M1 and NM plants (**Figure 5C**). In contrast in root GSSG showed decrease with increasing levels of As stress. However, at low levels of As stress (0 and 25 mg As kg^-1^ soil), GSSG concentration was higher in M plants (**Figure 5D**). At higher levels (50 and 100 mg As kg^-1^ soil), the effect of the two AMF species was variable.

In shoot as well as root of NM plants, the GSH:GSSG ratio decreased with increase in As concentration. In M plants (M1 and M2) there was not a definite pattern in variation of GSH:GSSG ratio, although their value remained higher than NM equivalents (**Figures 5E,F**).

Two-way ANOVA revealed significant influence of As, AMF and interactive effects of As × AMF treatment on GR activity in shoot as well as root (**Table 1**). Activity of GR enhanced on increasing the concentration of As in NM, M1, and M2 plants, however, GR activity was more in M1 and M2 plants as compared to NM plants (**Figures 5G,H**). GR activity and GSH concentration in shoot showed moderate positive correlation, however, these showed weak negative correlation in root (GR-GSH: *r*_shoot_ = -0.61; *r*_root_ = -0.41).

### Glyoxalase System

**FIGURE 6:**
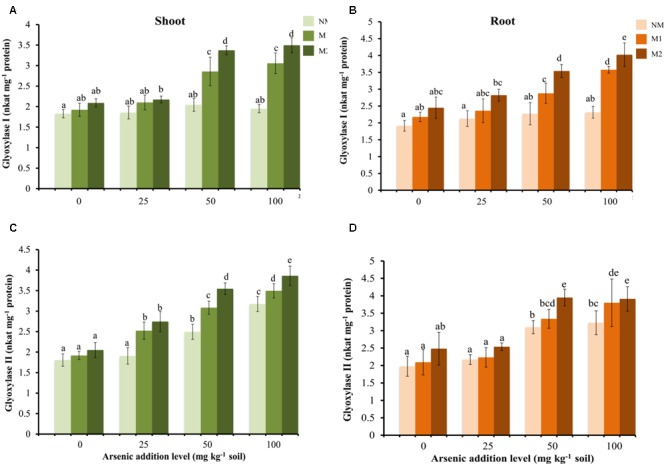
Effect of As addition levels and AMF treatments on the activities of Gly I in **(A)** shoot and **(B)** root and Gly II in **(C)** shoot and **(D)** root of *T. aestivum* L. var. HD-2967 plants. Data is represented as mean ± SD (*n* = 5). Bars showing different letters indicate significant differences among treatments according to the Duncan’s multiple comparison test (*p* < 0.05); where NM, non-mycorrhizal; M1, *R. intraradices*; M2, *G. etunicatum.*

No significant increase was found in Gly I activity in shoot and root of NM plants at all concentrations of As (**Figures 6A,B**). While in M plants Gly I activity increased at higher concentrations of As (50 and 100 mg kg^-1^ soil).

The activity of Gly II increased with increase in the concentration of As in shoots of both NM and M plants (**Figure 6C**). However, the increase was non-significant between 0 and 25 mg As kg^-1^ soil for NM plants. Mycorrhizal plants exhibited higher enzyme activity at all levels of As corresponding to NM plants. In roots the activity of Gly II exhibited no significant increase at 25 mg As kg^-1^ soil but increased significantly at 50 and 100 mg As kg^-1^ soil in both NM and M plants with higher increase of the enzyme activity in M plants (**Figure 6D**). Two-way analysis revealed significant effects of As and AMF treatments independently and by their interaction on Gly I activity both in shoot and root, and Gly II activity in shoot. However, Gly II activity was significantly influenced by As and AMF in root but remained unaffected by their interaction (**Table 1**).

### Thiol Metabolites

**FIGURE 7:**
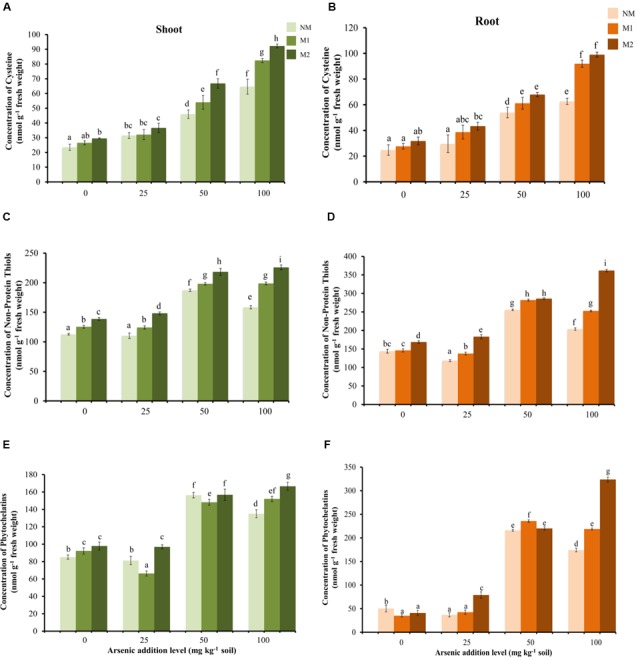
Effect of As addition levels and AMF treatments on concentrations of cysteine in **(A)** shoot and **(B)** root, NPSHs in **(C)** shoot and **(D)** root and PCs in **(E)** shoot and **(F)** root of *T. aestivum* L. var. HD-2967 plants. Data is represented as mean ± SD (*n* = 5). Bars showing different letters indicate significant differences among treatments according to the Duncan’s multiple comparison test (*p* < 0.05); where NM, non-mycorrhizal; M1, *R. intraradices*; M2, *G. etunicatum.*

Cysteine and NPSHs were significantly influenced by As and AMF treatments independently as well as by their interaction (**Table 1**). Cysteine showed a gradual rise in shoot and root with increase in As in soil in all the treatments (**Figures 7A,B**). The positive influence of AMF inoculation was apparent in higher levels of As (50 and 100 mg kg^-1^) additions. Concentration of NPSHs increased with rise in As from 25 to 50 mg As kg^-1^ soil, a further surge in As exposure to 100 mg As kg^-1^ soil resulted in sharp decline in NM plants (root and shoot) (**Figures 7C,D**). In M2 plants the level of NPSHs remained maximum at all levels of As stress. The concentration of PCs increased with increase in As from 25 to 50 mg kg^-1^ soil in all treatments (**Figures 7E,F**). However, positive effect of AMF inoculation was evident distinctly only at 100 mg As kg^-1^ soil. Another interesting observation is that the concentrations of thiol metabolites are higher in root in comparison to shoot.

**FIGURE 8:**
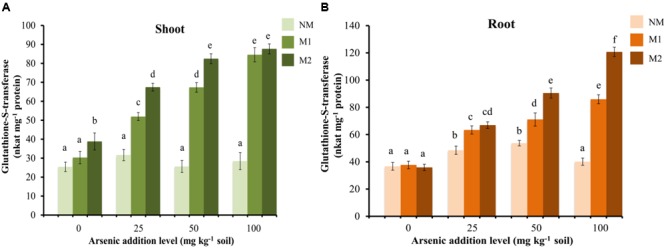
Effect of As addition levels and AMF treatments on the activity of GST in **(A)** shoot and **(B)** root of *T. aestivum* L. var. HD-2967 plants. Data is represented as mean ± SD (*n* = 5). Bars showing different letters indicate significant differences among treatments according to the Duncan’s multiple comparison test (*p* < 0.05); where NM, non-mycorrhizal; M1, *R. intraradices*; M2, *G. etunicatum.*

The GST activity showed no significant effect in shoot of NM plants, while it enhanced with increase in the concentration of As in the soil in M plants (**Figure 8A**). In root the enzyme activity increased significantly in M plants while, in NM plant there was increase in the activity up to 50 mg As kg^-1^ soil (**Figure 8B**). Beyond this it declined. The activity of GST was significantly influenced by treatments of As and AMF independently as well as by their interaction in both shoot and root (**Table 1**).

## Discussion

Arsenic additions had variable effects on the percentage of *T. aestivum* root colonized by *R. intraradices* and *G. etunicatum.* The reports on effect of As on percent of root colonized are not consistent. While many experiments have reported no decrease in percent colonization when As was artificially added to soil (Trotta et al., 2006; Chen et al., 2007; Christophersen et al., 2009), there are reports of reduction in colonization (Liu et al., 2005; Garg et al., 2015), and also an increase (Al Agely et al., 2005). In spite of this difference in root colonization with respect to As, each mycobiont conferred benefits to the host plant. Nevertheless the extent of assistance varied with AMF species participating in the formation of AM, and the parameter assayed. At each level of As in soil, M plants continued to display better growth than NM plants, suggesting alleviation of As toxicity.

Root and shoot As concentrations in wheat plants increased proportionately to the level of As added to the soil. However, As concentrations were higher in tissues (root, shoot, and grain) of NM plants than M plants. The greater biomass has ensued dilution of the harmful metalloid in plant tissues, limiting its toxic effect. AM mediated significant dilution of As in plant tissues has been previously reported (Liu et al., 2005; Chen et al., 2007; Cozzolino et al., 2010). In contrast at higher levels of As stress, the content of As in M plant tissue (shoot and root) was higher than NM plants. Reports on total As content in AM plants are variable, with values higher than in NM plants observed in both roots and shoots of maize (Xia et al., 2007) and lower values in sunflower and lentil (Ahmed et al., 2006; Ultra et al., 2007). Increased total As content in AM plants may be the result of more extensive root systems in the larger plants that make an important contribution on a whole plant basis (Chen et al., 2007).

Since arsenate [As(V)] and inorganic phosphate (Pi) have similar properties, it is expected that there exists a competition between As(V) and Pi in all cellular processes. Foremost, As(V) competes with Pi for entry (Meharg, 1994; Zhao et al., 2010). Thereafter, cytoplasmic As(V) contends for phosphate binding sites and hence inhibits metabolic processes (Geng et al., 2006; Smith et al., 2010a; Srivastava and Sharma, 2013). Therefore, maintenance of a high cytosolic P:As ratio is necessary in order to enhance plant As tolerance. In this study, P concentration in plant tissues decreased as the As concentration in the soil increased causing an As-induced P deficiency. This may be one of the crucial reasons accounting for the reduction in growth of *T. aestivum* plants under As stress observed in the study. However, our observation that AMF-colonized plants resulted in higher shoot and root P concentrations compared to NM plants in As spiked soil, shows the potential of AM in preserving the P homeostasis. These results are in accordance to earlier reports wherein AM plants displayed selective uptake and transfer of P over As (Chen et al., 2007; Dong et al., 2008). The capacity of M plants to sustain a high P:As ratio is one of the key reasons for plant tolerance to As toxicity (Liu et al., 2005; Chen et al., 2007; Cozzolino et al., 2010).

The AM pathway involves Pi uptake by the external hyphae and translocation to the plant through fungal hyphae and transfer across specialized symbiotic interfaces in root cortical cells (Smith and Read, 2008). This pathway overcomes severe diffusion limitation, bypasses the sharp depletion zones close to roots and delivers Pi direct to root cortical cells (Smith and Read, 2008). In addition, fungal translocation of P is extremely rapid, and involves movement of polyphosphate from sites of Pi absorption to sites of breakdown and transfer to plant cells (Ezawa et al., 2002). Since polyarsenate is not formed (due to low stability), As(V) cannot be translocated in the same way. Furthermore establishment of the AM pathway is commonly accompanied by a decreased contribution of the direct pathway (Smith et al., 2010a; Chen et al., 2013).

Separate Pi transporters play key roles in the direct and AM uptake pathways, with AM-inducible plant transporters expressed in colonized cells in the root cortex of plants (Smith et al., 2003; Bucher, 2007; Javot et al., 2007). In rice effect of mycorrhizal inoculation on phosphate transporters (OsPTs) under As stress has been studied in detail. Arsenate uptake is reported to be restricted as the expressions of 6 out of 11 phosphate transporters in rice roots were decreased upon mycorrhizal symbiosis (Paszkowski et al., 2002). AMF are able to assist the plant to obtain phosphate by inducing OsPT11, which transport Pi between cells rather than between outside media and cell (Glassop et al., 2005; Chen et al., 2013). Therefore, it is assumed that mycorrhiza-specific Pi uptake system controls the direct uptake system and helps the wheat to take up more phosphate and less arsenate, resulting in higher P:As ratio observed in this study. Nevertheless, more study on effect of AMF inoculation on phosphate transporters of wheat (*T. aestivum*) under different levels of As(V) stress is required to confirm this.

Formation of AM in wheat roots influenced distribution of As in plant tissues. The TF increased at higher concentrations of As, contradictory to the observations of Dong et al. (2008). An enhancement in As TF in M plants may presumably be due to larger magnitude of decline in As concentration in root as compared with shoots. Also supported by an increase in the As storing capacity of the leaves (by virtue of increased biomass) and/or As exclusion in root (Trotta et al., 2006). The latter is also supported by low bioaccumulation values observed in M plants at high As addition. However, further As translocation to wheat grain was reduced as evident by lower As grain:shoot ratio in M plants compared with NM plants at each level of As stress. Less As accumulation in grain would have immense significance with respect to As toxicity in food chain.

Arsenate uptake triggered oxidative stress resulting in increased generation of H_2_O_2_ and lipid peroxidation as reported in earlier studies (Singh et al., 2007; Liu et al., 2013). Interestingly, the levels of oxidative stress were much higher in roots than in shoots. This observation supports the view that most of the As(V) taken up by the plant is reduced to As(III) in the roots (Smith et al., 2010a). To cope with enhanced levels of oxidative stress, plants are equipped with antioxidant system that gets activated under As stress (Singh et al., 2007; Sobrino-Plata et al., 2014). APX, GPX, and CAT play a crucial role in H_2_O_2_ degradation. Under As stress, activities of these enzymes were enhanced due to a higher level of H_2_O_2_. This is in agreement with our results where the activities of antioxidant enzymes (SOD, CAT, GPX, and APX) and concentration of α-tocopherols increased with concentration of As in soil.

The present study demonstrates a reduction in As-induced oxidative damage in wheat plants colonized by *R. intraradices* or *G. etunicatum*. The alleviation potential of AM was more evident with increase in severity of As stress. Mycorrhizal plants displayed lower lipid peroxidation and H_2_O_2_ levels than NM plants. Several studies have shown that when subjected to stress, lipid peroxidation is lesser in M than the NM plants indicating that AMF inoculation helps in reduction of oxidative damage (Evelin and Kapoor, 2014; Tan et al., 2015; Yang et al., 2015; Jiang et al., 2016). It is widely accepted that diminishing H_2_O_2_ levels is one of the strategies by which AM protects plants against diverse stresses (Hajiboland et al., 2010; Ruiz-Sánchez et al., 2010; Garg and Bhandari, 2012). Recently, it has been observed that H_2_O_2_ is effusive in roots and there was a higher net H_2_O_2_ efflux in roots when colonized by AMF via aquaporin channels present on the membranes of external hyphae (Zou et al., 2015).

A better ROS scavenging system in M wheat plants is observed in accordance with earlier studies (Garg and Kaur, 2013; Garg and Chandel, 2015). Alpha-tocopherols disrupt the chain propagation step in lipid auto-oxidation (Serbinonva and Packer, 1994), while carotenoids protect photosynthetic apparatus by quenching ROS (Collins, 2001). Proline is reported to inhibit the apoptotic responses triggered by a variety of abiotic stresses by scavenging intracellular hydroxyl radical, in addition to its well-established role as an osmolyte (Chen and Dickman, 2005; Ozden et al., 2009; Gill and Tuteja, 2010; Carvalho et al., 2015). Enhanced proline synthesis plays an important role in refilling NADPH to maintain GSH and ASA in the reduced state by potentiating pentose-phosphate pathway activity (Verbruggen and Hermans, 2008). Under all As levels, AMF colonization consistently increased concentrations of the antioxidant molecules α-tocopherol and carotenoids while the concentration of proline was more in M plants at high As level. Higher concentration of these antioxidants contribute to drop in lipid peroxidation in M plants.

Superoxide dismutase is a metalloenzyme and exists in different isoforms based on the metal cofactor: iron (Fe-SOD), manganese (Mn-SOD), and copper–zinc (Cu–Zn SOD; Alscher et al., 2002). The activity of each SOD isoform is influenced by the availability of respective co-factor (Alguacil et al., 2003). Similarly, CAT and APX are metalloenzymes, and their enzymatic activity is dependent on the availability of their cofactors. Enhanced activity of SOD, CAT, and APX may be due to higher uptake of these micronutrients in M plants. Further SOD, CAT, and GPX transcripts or enzymatic activity often increase in response to As exposure (Mylona et al., 1998; Stoeva and Bineva, 2003; Srivastava et al., 2005; Abercrombie et al., 2008; Ahsan et al., 2008; Norton et al., 2008; Chakrabarty et al., 2009). This explains induction of these enzyme activities in NM plants in the present study also. However, the isoforms of the various enzymes are differentially expressed in response to As(V) and As(III) (Finnegan and Chen, 2012). It is known that AMF colonization has considerable effect on the composition of As species and their accumulation in plant (Zhang et al., 2014). The changes in the activities of antioxidant enzymes in M plants may be due to differential influence on different isoforms as a result of alteration in As speciation and their cellular distribution. However, the preparation procedure of the samples for enzymatic assays does not include organellar isoenzymes in this study. Further investigations are required to validate this.

In plants ascorbate-glutathione (AsA-GSH) pathway is the second main component for neutralizing H_2_O_2_. The AsA-GSH antioxidant defense pathway consists of both enzymatic and non-enzymatic antioxidants. The accumulation of ascorbate is one of the main effects of As(V) uptake (Srivastava et al., 2005; Singh et al., 2006; Khan et al., 2009). In this study also, increase in As(V) in soil stimulated accumulation of ascorbate in wheat plants. However, the concentration of AsA was higher in M plants compared with NM plants. AM fungal colonization resulted in higher activities of DHAR and MDHAR in comparison with NM plants, and thus explains a higher level of AsA in M plants. Mycorrhizal plants upheld a higher P:As ratio and consequently prevent replacement of Pi by As(V) during photophosphorylation for the synthesis of ATP. Consequently As-induced adverse effects on photosynthesis get alleviated in M plants. Hence higher concentration of AsA may also be due to more primary metabolites available for its synthesis in M plants.

The GSH:GSSG ratio plays an important role in maintaining the redox state of the cell and, GR plays a crucial role in maintaining the GSH:GSSG ratio by catalyzing reduction of GSSG to GSH (Dixit et al., 2015a). As addition levels led to a significant increase in GSH as well as GSSG concentrations in shoots of wheat, while the ratio of GSH:GSSG decreased. These results are consistent with the observations of Hasanuzzaman and Fujita (2013). Despite of the enhanced levels of GSSG and stimulation of GR activity in NM plants up to 50 mg As kg^-1^ soil, GSH:GSSG declined. It suggests that As triggered activation of GR was not adequate to overcome GSH consumption in As(III) complexation (Jozefczak et al., 2012). AM appeared to maintain a favorable GSH:GSSG ratio due to, (i) decreased oxidative stress owing to the reduced concentration of As in plant tissue (root as well as shoot); (ii) better ability to detoxify H_2_O_2_ by other components of oxidative defense system (higher enzymatic and non-enzymatic antioxidants) hence relieving pressure on GSH to maintain cellular redox state; (iii) more biosynthesis of GSH.

The glyoxalase system prevents buildup of MG, and converts it to non-toxic hydroxyacids such as lactate. It consists of two enzymes (Gly I and Gly II) acting in concert. The upregulation or overexpression of these enzymes has been reported to impart tolerance to abiotic stresses (Singla-Pareek et al., 2008; Saxena et al., 2011). In this study, M plants demonstrated higher activities of Gly I and Gly II at any level of As than NM plants which suggested more efficient detoxification of MG in M plants. These results are in agreement with observations of Shamshiri and Fattahi (2014) where M plants showed lower MG concentration than NM plants under salt stress. Furthermore, a few studies have reported higher expression of Gly I in M plants (Campos-Soriano et al., 2011; Fan and Liu, 2011).

Detoxification mechanisms for As(III) include efflux from the roots, sequestration in cell vacuoles and complexation with thiols (PCs and glutathione) for which As(III) has very high-affinity. Glutathione protects the cell from free metal ions by forming non-toxic complexes and facilitates their sequestration while the enzyme GST catalyzes these conjugations (Jozefczak et al., 2012). Uptake of As induced the increase in concentration of thiol metabolites (GSH, NPSHs, and PCs) and incited GST activity suggesting inherent As tolerance in the wheat cultivar used in this study. However, AMF colonization (both M1 or M2) further increased the concentrations of these metabolites and GST activity, suggesting more capacity in M plants to sequester As also reflected by higher As content in M roots than NM roots.

The biosynthesis of GSH (and PCs) requires adequate supplies of glutamine, cysteine, and glycine. Among these, cysteine is by far the limiting substrate for GSH biosynthesis (Noctor et al., 1998). Increase in concentration of cysteine in NM and M plants explains enhanced concentration of GSH, NPSHs, and PCs. Further higher levels of cysteine in M than NM plants resulted in higher concentration of GSH, NPSHs, and PCs in them. Several studies have reported effect of S supply on As uptake, translocation and accumulation in plants (Fan et al., 2013; Dixit et al., 2015a,b; Srivastava et al., 2016). Concomitant studies on AM have shown that in addition to other nutrients (N, P), sulfur (S) compounds are symbiotically transferred from AM fungus to host plants (Casieri et al., 2012; Sieh et al., 2013). Symbiosis by AMF contributes to plant’s S nutrition by transport of S, as well as organic S-containing compounds (such as cysteine, methionine, and glutathione), via the mycorrhizal pathway (Allen and Shachar-Hill, 2009). Therefore, it is tempting to propose here that the higher concentrations of the thiol compounds observed in the present study may be due to enhanced uptake and assimilation of S in M over NM plants. However, more studies are needed to relate S metabolism in M plants with As tolerance.

The present study demonstrates an increased As tolerance in mycorrhizal wheat plants. The study reveals multifarious role of AMF in alleviation of As toxicity. Formation of AM has systemic effect on the physiology and biochemistry of the host plants. The effects of *R. intraradices* and *G. etunicatum* in alleviation of As stress were largely same. However, extent of response varied with the level of As stress, participating mycobiont and the parameter analyzed. Higher As tolerance in M plants (i) is not due to decreased up take of As but due to dilution effect as a result of more biomass; (ii) more favorable P:As ratio; (iii) a better antioxidative capacity; (iv) augmented glyoxalase system, and (v) higher thiol metabolites to sequester As. However, more studies are required to decipher the physiological and molecular basis of changes in antioxidant system and thiol metabolites in M plants under As-stress.

## Author Contributions

SS executed the experiments. GA and NS executed some of the experiments and helped in the preparation of the manuscript. RK designed the work, analyzed and interpreted the data, wrote the main manuscript. All authors reviewed and approved the manuscript.

## Conflict of Interest Statement

The authors declare that the research was conducted in the absence of any commercial or financial relationships that could be construed as a potential conflict of interest. The reviewer PCS and handling Editor declared their shared affiliation, and the handling Editor states that the process met the standards of a fair and objective review.

## Supplementary Material

FIGURE S1 Pictorial representation of various treatments of As and AMF innoculation in *Triticum aestivum L.* var. HD-2967.

FIGURE S2 Histochemical staining showing AMF colonization in root cortical cells of *Triticum aestivum L*. var. HD-2967 with **(A)** arbuscules and **(B)** vesicles of *Rhizoglomus intraradices*; **(C)** arbuscules and **(D)** vesicles of *Glomus etunicatum* stained with trypan blue. Bar = 50 μm.

## Abbreviations

AM: arbuscular mycorrhiza
AMF: arbuscular mycorrhizal fungi
APX: ascorbate peroxidase
As: arsenic
As(III): trivalent arsenite
As(V): trivalent arsenate
AsA: ascorbic acid
CAT: catalase
DHA: dehydroascorbic acid
DHAR: dehydroascorbate reductase
Gly I: glyoxalase I
Gly II: glyoxalase II
GPX: guaiacol peroxidase
GR: glutathione reductase
GSH: reduced glutathione
GSSG: glutathione disulphide
GST: glutathione-*S*-transferase
M: mycorrhizal
MDA: malondialdehyde
MDHAR: monodehydroascorbate reductase
NM: non-mycorrhizal
NPSHs: non-protein thiols
PCs: phytochelatins
ROS: reactive oxygen species
SOD: superoxide dismutase
TF: translocation factor

## References

[B1] Abercrombie J. M. Halfhill M. D. Ranjan P. Rao M. R. Saxton A. M. Yuan J. S. (2008). Transcriptional responses of *Arabidopsis thaliana* plants to As(V) stress. *BMC Plant Biol.* 8:87 10.1186/1471-2229-8-87 PMC2547109 18684332

[B2] Aebi H. Lester P. (1984). “Catalase in vitro,” in *Methods in Enzymology* Vol. 105 ed. Packer L. (New York, NY: Academic Press) 121–126. 10.1016/s0076-6879(84)05016-3 6727660

[B3] Ahmed F. R. S. Killham K. Alexander I. (2006). Influences of arbuscular mycorrhizal fungus *Glomus mosseae* on growth and nutrition of lentil irrigated with arsenic contaminated water. *Plant Soil* 283 33–41. 10.1007/s11104-005-0415-8

[B4] Ahsan N. Lee D. G. Alam I. Kim P. J. Lee J. J. Ahn Y. O. (2008). Comparative proteomic study of arsenic-induced differentially expressed proteins in rice roots reveals glutathione plays a central role during As stress. *Proteomics* 8 3561–3576. 10.1002/pmic.200701189 18752204

[B5] Al Agely A. Sylvia D. M. Ma L. Q. (2005). Mycorrhizae increase arsenic uptake by the hyperaccumulator Chinese brake fern (*Pteris vittata* L.). *J. Environ. Qual.* 34 2181–2186. 10.2134/jeq2004.0411 16275719

[B6] Alguacil M. M. Hernández J. A. Caravaca F. Portillo B. Roldan A. (2003). Antioxidant enzyme activities in shoots from three mycorrhizal shrub species afforested in a degraded semi-arid soil. *Physiol. Plant.* 118 562–570. 10.1034/j.1399-3054.2003.00149.x

[B7] Allen J. W. Shachar-Hill Y. (2009). Sulfur transfer through an arbuscular mycorrhiza. *Plant Physiol.* 149 549–560. 10.1104/pp.108.129866 18978070 PMC2613693

[B8] Allen S. E. (1989). *Chemical Analysis of Ecological Materials* 2nd Edn. Oxford: Blackwell Scientific Publications.

[B9] Alscher R. G. Erturk N. Heath L. S. (2002). Role of superoxide dismutases (SODs) in controlling oxidative stress in plants. *J. Exp. Bot.* 53 1331–1341. 10.1093/jexbot/53.372.1331 11997379

[B10] Anawar H. M. Akai J. Mostofa K. M. G. Safiullah S. Tareq S. M. (2002). Arsenic poisoning in groundwater: health risk and geochemical sources in Bangladesh. *Environ. Int.* 27 597–604. 10.1016/S0160-4120(01)00116-7 11871394

[B11] Arakawa N. Tsutsumi K. Sanceda N. G. Kurata T. Inagaki C. (1981). A rapid and sensitive method for the determination of ascorbic acid using 4, 7-diphenyl-l, 10-phenanthroline. *Agric. Biol. Chem.* 45 1289–1290. 10.1080/00021369.1981.10864697

[B12] Arnon D. I. (1949). Copper enzymes in isolated chloroplasts. Polyphenoloxidase in *Beta vulgaris*. *Plant Physiol.* 24 1–15. 10.1104/pp.24.1 16654194 PMC437905

[B13] Asati A. Pichhode M. Nikhil K. (2016). Effect of heavy metals on plants: an overview. *Int. J. Appl. Innov. Eng. Manage.* 5 2319–4847.

[B14] Bates L. S. Waldren R. P. Teare I. D. (1973). Rapid determination of free proline for water-stress studies. *Plant Soil* 39 205–207. 10.1007/BF00018060

[B15] Bhargava P. Srivastava A. K. Urmil S. Rai L. C. (2005). Phytochelatin plays a role in UV-B tolerance in N 2-fixing cyanobacterium *Anabaena doliolum*. *J. Plant Physiol.* 162 1220–1225. 10.1016/j.jplph.2004.12.006 16323273

[B16] Bhattacharya P. Samal A. C. Majumdar J. Santra S. C. (2010). Accumulation of arsenic and its distribution in rice plant (*Oryza sativa* L.) in Gangetic West Bengal, India. *Paddy Water Environ.* 8 63–70. 10.1007/s10333-009-0180-z

[B17] Bradford M. M. (1976). A rapid and sensitive method for the quantitation of microgram quantities of protein utilizing the principle of protein-dye binding. *Anal. Biochem.* 72 248–254. 10.1016/0003-2697(76)90527-3 942051

[B18] Bucher M. (2007). Functional biology of plant phosphate uptake at root and mycorrhiza interfaces. *New Phytol.* 173 11–26. 10.1111/j.1469-8137.2006.01935.x 17176390

[B19] Campos-Soriano L. García-Martínez J. Segundo B. S. (2011). The arbuscular mycorrhizal symbiosis promotes the systemic induction of regulatory defence-related genes in rice leaves and confers resistance to pathogen infection. *Mol. Plant Pathol.* 13 579–592. 10.1111/j.1364-3703.2011.00773.x 22212404 PMC6638712

[B20] Carvalho L. C. Vidigal P. Amâncio S. (2015). Oxidative stress homeostasis in grapevine (*Vitis vinifera* L.). *Front. Environ. Sci.* 3:20 10.3389/fenvs.2015.00020

[B21] Casieri L. Gallardo K. Wipf D. (2012). Transcriptional response of *Medicago truncatula* sulphate transporters to arbuscular mycorrhizal symbiosis with and without sulphur stress. *Planta* 235 1431–1447. 10.1007/s00425-012-1645-7 22535379

[B22] Chakrabarty D. Trivedi P. K. Misra P. Tiwari M. Shri M. Shukla D. (2009). Comparative transcriptome analysis of arsenate and arsenite stresses in rice seedlings. *Chemosphere* 74 688–702. 10.1016/j.chemosphere.2008.09.082 18996570

[B23] Chatterjee J. Chatterjee C. (2000). Phytotoxicity of cobalt, chromium and copper in cauliflower. *Environ. Pollut.* 109 69–74. 10.1016/S0269-7491(99)00238-9 15092914

[B24] Chen B. Xiao X. Zhu Y. G. Smith F. A. Xie Z. M. Smith S. E. (2007). The arbuscular mycorrhizal fungus *Glomus mosseae* gives contradictory effects on phosphorus and arsenic acquisition by *Medicago sativa* L. *Sci. Total Environ.* 379 226–234. 10.1016/j.scitotenv.2006.07.038 17157359

[B25] Chen C. Dickman M. B. (2005). Proline suppresses apoptosis in the fungal pathogen *Colletotrichum trifolii*. *Proc. Natl. Acad. Sci. U.S.A.* 102 3459–3464. 10.1073/pnas.0407960102 15699356 PMC552905

[B26] Chen X. W. Wu F. Y. Li H. Chan W. F. Wu C. Wu S. C. (2013). Phosphate transporters expression in rice (*Oryza sativa* L.) associated with arbuscular mycorrhizal fungi (AMF) colonization under different levels of arsenate stress. *Environ. Exp. Bot.* 87 92–99. 10.1016/j.envexpbot.2012.08.002 22944255

[B27] Chowdhury U. K. Biswas B. K. Chowdhury T. R. Samanta G. Mandal B. K. Basu G. C. (2000). Groundwater arsenic contamination in Bangladesh and West Bengal. India. *Environ. Health Perspect.* 108 393–397. 10.2307/3454378 10811564 PMC1638054

[B28] Christophersen H. M. Smith F. A. Smith S. E. (2009). Arbuscular mycorrhizal colonization reduces arsenate uptake in barley via downregulation of transporters in the direct epidermal phosphate uptake pathway. *New Phytol.* 184 962–974. 10.1111/j.1469-8137.2009.03009.x 19754635

[B29] Collins A. (2001). Carotenoids and genomic stability. *Mutat. Res.* 475 21–28. 10.1016/S0027-5107(01)00071-9 11295150

[B30] Cox M. S. Kovar J. L. (2001). Soil arsenic effects on canola seedling growth and ion uptake. *Commun. Soil Sci. Plant Anal.* 32 107–117. 10.1081/CSS-100102996

[B31] Cozzolino V. Pigna M. Di Meo V. Caporale A. G. Violante A. (2010). Effects of arbuscular mycorrhizal inoculation and phosphorus supply on the growth of *Lactuca sativa* L. and arsenic and phosphorus availability in an arsenic polluted soil under non-sterile conditions. *Appl. Soil Ecol.* 45 262–268. 10.1016/j.apsoil.2010.05.001

[B32] Dixit G. Singh A. P. Kumar A. Dwivedi S. Deeba F. Kumar S. (2015a). Sulfur alleviates arsenic toxicity by reducing its accumulation and modulating proteome, amino acids and thiol metabolism in rice leaves. *Sci. Rep.* 5:16205 10.1038/srep16205 PMC4639781 26552588

[B33] Dixit G. Singh A. P. Kumar A. Singh P. K. Kumar S. Dwivedi S. (2015b). Sulfur mediated reduction of arsenic toxicity involves efficient thiol metabolism and the antioxidant defense system in rice. *J. Hazard. Mater.* 298 241–251. 10.1016/j.jhazmat.2015.06.008 26073379

[B34] Dong Y. Zhu Y. G. Smith F. A. Wang Y. Chen B. (2008). Arbuscular mycorrhiza enhanced arsenic resistance of both white clover (*Trifolium repens* L.) and ryegrass (*Lolium perenne* L.) plants in an arsenic-contaminated soil. *Environ. Pollut.* 155 174–181. 10.1016/j.envpol.2007.10.023 18060670

[B35] Egley G. H. Paul R. N. Jr. Vaughn K. C. Duke S. O. (1983). Role of peroxidase in the development of water-impermeable seed coats in *Sida spinosa* L. *Planta* 157 224–232. 10.1007/BF00405186 24264151

[B36] Elavarthi S. Martin B. (2010). Spectrophotometric assays for antioxidant enzymes in plants. *Methods Mol. Biol.* 639 273–280. 10.1007/978-1-60761-702-0_16 20387052

[B37] Ellman G. L. (1959). Tissue sulfhydryl groups. *Arch. Biochem. Biophys.* 82 70–77. 10.1016/0003-9861(59)90090-6 13650640

[B38] Evelin H. Kapoor R. (2014). Arbuscular mycorrhizal symbiosis modulates antioxidant response in salt-stressed *Trigonella foenum-graecum* plants. *Mycorrhiza* 24 197–208. 10.1007/s00572-013-0529-4 24113907

[B39] Ezawa T. Smith S. E. Smith F. A. (2002). P metabolism and transport in AM fungi. *Plant Soil* 244 221–230. 10.1023/A:1020258325010

[B40] Fan J. Xia X. Hu Z. Ziadi N. Liu C. (2013). Excessive sulfur supply reduces arsenic accumulation in brown rice. *Plant Soil Environ.* 59 169–174. 10.1016/j.envpol.2009.08.042 19781829

[B41] Fan Q. J. Liu J. H. (2011). Colonization with arbuscular mycorrhizal fungus affects growth, drought tolerance and expression of stress-responsive genes in *Poncirus trifoliata*. *Acta Physiol. Plant.* 33 1533–1542. 10.1007/s11738-011-0789-6

[B42] Finnegan P. Chen W. (2012). Arsenic toxicity: the effects on plant metabolism. *Front. Physiol.* 3:182 10.3389/fphys.2012.00182 PMC3368394 22685440

[B43] Gaitonde M. K. (1967). A spectrophotometric method for the direct determination of cysteine in the presence of other naturally occurring amino acids. *Biochem. J.* 104 627–633. 10.1042/bj1050897b 6048802 PMC1270629

[B44] Garg N. Bhandari P. (2012). Influence of cadmium stress and arbuscular mycorrhizal fungi on nodule senescence in *Cajanus cajan* (L.) Mill sp. *Int. J. Phytoremediation* 14 62–74. 10.1080/15226514.2011.573822 22567695

[B45] Garg N. Chandel S. (2015). Role of arbuscular mycorrhiza in arresting reactive oxygen species (ROS) and strengthening antioxidant defense in *Cajanus cajan* (L.) Mill sp. nodules under salinity (NaCl) and cadmium (Cd) stress. *Plant Growth Regul.* 75 521–534. 10.1007/s10725-014-0016-8

[B46] Garg N. Kaur H. (2013). Response of antioxidant enzymes, phytochelatins and glutathione production towards Cd and Zn stresses in *Cajanus cajan* (L.) Mill sp. genotypes colonized by arbuscular mycorrhizal fungi. *J. Agron. Crop Sci.* 199 118–133. 10.1111/j.1439-037X.2012.00533.x

[B47] Garg N. Singla P. (2012). The role of *Glomus mosseae* on key physiological and biochemical parameters of pea plants grown in arsenic contaminated soil. *Sci. Hortic.* 143 92–101. 10.1016/j.scienta.2012.06.010

[B48] Garg N. Singla P. Bhandari P. (2015). Metal uptake, oxidative metabolism, and mycorrhization in pigeonpea and pea under arsenic and cadmium stress. *Turk. J. Agric. For.* 39 234–250. 10.3906/tar-1406-121

[B49] Geng C. N. Zhu Y. G. Hu Y. Williams P. Meharg A. A. (2006). Arsenate causes differential acute toxicity to two P-deprived genotypes of rice seedlings (*Oryza sativa* L.). *Plant Soil* 279 297–306. 10.1007/s11104-005-1813-7

[B50] Gill S. S. Tuteja N. (2010). Reactive oxygen species and antioxidant machinery in abiotic stress tolerance in crop plants. *Plant Physiol. Biochem.* 48 909–930. 10.1016/j.plaphy.2010.08.016 20870416

[B51] Giovannetti M. Mosse B. (1980). An evaluation of techniques for measuring vesicular arbuscular mycorrhizal infection in roots. *New Phytol.* 84 489–500. 10.1111/j.1469-8137.1980.tb04556.x

[B52] Glassop D. Smith S. E. Smith F. W. (2005). Cereal phosphate transporters associated with the mycorrhizal pathway of phosphate uptake into roots. *Planta* 222 688–698. 10.1007/s00425-005-0015-0 16133217

[B53] Gonzalez-Chavez C. Harris P. J. Dodd J. Meharg A. A. (2002). Arbuscular mycorrhizal fungi confer enhanced arsenate resistance on *Holcus lanatus*. *New Phytol.* 155 163–171. 10.1046/j.1469-8137.2002.00430.x 33873289

[B54] Gupta R. Seth A. (2007). A review of resource conserving technologies for sustainable management of the rice–wheat cropping systems of the Indo-Gangetic plains (IGP). *Crop Prot.* 26 436–447. 10.1016/j.cropro.2006.04.030

[B55] Habig W. H. Jakoby W. B. (1981). Assays for differentiation of glutathione S-transferases. *Methods Enzymol.* 77 398–405. 10.1016/S0076-6879(81)77053-8 7329316

[B56] Hajiboland R. Aliasgharzadeh N. Laiegh S. F. Poschenrieder C. (2010). Colonization with arbuscular mycorrhizal fungi improves salinity tolerance of tomato (*Solanum lycopersicum* L.) plants. *Plant Soil* 331 313–327. 10.1007/s11104-009-0255-z

[B57] Hasanuzzaman M. Fujita M. (2013). Exogenous sodium nitroprusside alleviates arsenic-induced oxidative stress in wheat (*Triticum aestivum* L.) seedlings by enhancing antioxidant defense and glyoxalase system. *Ecotoxicology* 22 584–596. 10.1007/s10646-013-1050-4 23430410

[B58] Heath R. L. Packer L. (1968). Photoperoxidation in isolated chloroplasts: I. Kinetics and stoichiometry of fatty acid peroxidation. *Arch. Biochem. Biophys.* 125 189–198. 10.1016/0003-9861(68)90654-1 5655425

[B59] Hoque T. S. Hossain M. A. Mostofa M. G. Burritt D. J. Fujita M. Tran L. S. P. (2016). Methylglyoxal: an emerging signaling molecule in plant abiotic stress responses and tolerance. *Front. Plant Sci.* 7:1341 10.3389/fpls.2016.01341 PMC5020096 27679640

[B60] Hossain M. A. Hossain M. Z. Fujita M. (2009). Stress-induced changes of methylglyoxal level and glyoxalase I activity in pumpkin seedlings and cDNA cloning of glyoxalase I gene. *Aust. J. Crop Sci.* 3 53–64.

[B61] Huang R. Q. Gao S. F. Wang W. L. Staunton S. Wang G. (2006). Soil arsenic availability and the transfer of soil arsenic to crops in suburban areas in Fujian Province, southeast China. *Sci. Total Environ.* 368 531–541. 10.1016/j.scitotenv.2006.03.013 16624379

[B62] Javot H. Pumplin N. Harrison M. J. (2007). Phosphate in the arbuscular mycorrhizal symbiosis: transport properties and regulatory roles. *Plant Cell Environ.* 30 310–322. 10.1111/j.1365-3040.2006.01617.x 17263776

[B63] Jiang Q. Y. Zhuo F. Long S. H. Zhao H. D. Yang D. J. Ye Z. H. (2016). Can arbuscular mycorrhizal fungi reduce Cd uptake and alleviate Cd toxicity of *Lonicera japonica* grown in Cd-added soils? *Sci. Rep.* 6:21805 10.1038/srep21805 PMC4759589 26892768

[B64] Jozefczak M. Remans T. Vangronsfeld J. Cuypers A. (2012). Glutathione is a key player in metal-induced oxidative stress defenses. *Int. J. Mol. Sci.* 13 3145–3175. 10.3390/ijms13033145 22489146 PMC3317707

[B65] Kapoor R. Giri B. Mukerji K. G. (2002). *Glomus macrocarpum*: a potential bioinoculant to improve essential oil quality and concentration in Dill (*Anethum graveolens* L.) and Carum (*Trachyspermum ammi* (L.) Sprague). *World J. Microbiol. Biotechnol.* 18 459–463. 10.1023/A:1015522100497

[B66] Khan I. Ahmad A. Iqbal M. (2009). Modulation of antioxidant defense system for arsenic detoxification in Indian mustard. *Ecotoxicol. Environ. Saf.* 72 626–634. 10.1016/j.ecoenv.2007.11.016 18262648

[B67] Li H. Chen X. W. Wong M. H. (2016). Arbuscular mycorrhizal fungi reduced the ratios of inorganic/organic arsenic in rice grains. *Chemosphere* 145 224–230. 10.1016/j.chemosphere.2015.10.067 26688259

[B68] Liu J. Macarisin D. Wisniewski M. Sui Y. Droby S. Norelli J. (2013). Production of hydrogen peroxide and expression of ROS-generating genes in peach flower petals in response to host and non-host fungal pathogens. *Plant Pathol.* 62 820–828. 10.1111/j.1365-3059.2012.02683.x

[B69] Liu Q. J. Zheng C. M. Hu C. X. Tan Q. L. Sun X. C. Su J. J. (2012). Effects of high concentrations of soil arsenic on the growth of winter wheat (*Triticum aestivum* L) and rape (*Brassica napus*). *Plant Soil Environ.* 58 22–27.

[B70] Liu Y. Zhu Y. G. Chen B. D. Christie P. Li X. L. (2005). Yield and arsenate uptake of arbuscular mycorrhizal tomato colonized by *Glomus mosseae* BEG167 in As spiked soil under glasshouse conditions. *Environ. Int.* 31 867–873. 10.1016/j.envint.2005.05.041 15982738

[B71] Meharg A. A. (1994). Integrated tolerance mechanisms: constitutive and adaptive plant responses to elevated metal concentrations in the environment. *Plant Cell Environ.* 17 989–993. 10.1111/j.1365-3040.1994.tb02032.x

[B72] Meharg A. A. Cairney J. W. (1999). Co-evolution of mycorrhizal symbionts and their hosts to metal-contaminated environments. *Adv. Ecol. Res.* 30 69–112. 10.1016/S0065-2504(08)60017-3

[B73] Meharg A. A. Hartley-Whitaker J. (2002). Arsenic uptake and metabolism in arsenic resistant and nonresistant plant species. *New Phytol.* 154 29–43. 10.1046/j.1469-8137.2002.00363.x

[B74] Meharg A. A. Williams P. N. Adomako E. Lawgali Y. Y. Deacon C. Villada A. (2009). Geographical variation in total and inorganic arsenic content of polished (white) rice. *Environ. Sci. Technol.* 43 1612–1617. 10.1021/es802612a 19350943

[B75] Mishra S. Tripathi R. D. Srivastava S. Dwivedi S. Trivedi P. K. Dhankher O. P. (2009). Thiol metabolism play significant role during cadmium detoxification by *Ceratophyllum demersum* L. *Bioresour. Technol.* 100 2155–2161. 10.1016/j.biortech.2008.10.041 19091554

[B76] Miyake C. Asada K. (1992). Thylakoid-bound ascorbate peroxidase in spinach chloroplasts and photoreduction of its primary oxidation product monodehydroascorbate radicals in thylakoids. *Plant Cell Physiol.* 33 541–553. 10.1093/oxfordjournals.pcp.a078288

[B77] Moreno-Jiménez E. Esteban E. Peñalosa J. M. (2012). The fate of arsenic in soil-plant systems. *Rev. Environ. Contam. Toxicol.* 215 1–37. 10.1007/978-1-4614-1463-6_1 22057929

[B78] Mylona P. V. Polidoros A. N. Scandalios J. G. (1998). Modulation of antioxidant responses by arsenic in maize. *Free Radic. Biol. Med.* 25 576–585. 10.1016/S0891-5849(98)00090-2 9741595

[B79] Nakagawara S. Sagisaka S. (1984). Increase in enzyme activities related to ascorbate metabolism during cold acclimation in poplar twigs. *Plant Cell Physiol.* 25 899–906. 10.1093/oxfordjournals.pcp.a076804

[B80] Nakano Y. Asada K. (1981). Hydrogen peroxide is scavenged by ascorbate-specific peroxidase in spinach chloroplasts. *Plant Cell Physiol.* 22 867–880. 10.1093/oxfordjournals.pcp.a076232

[B81] Noctor G. Arisi A. C. M. Jouanin L. Kunert K. J. Rennenberg H. Foyer C. H. (1998). Glutathione: biosynthesis, metabolism and relationship to stress tolerance explored in transformed plants. *J. Exp. Bot.* 49 623–647. 10.1093/jxb/49.321.623

[B82] Norra S. Berner Z. A. Agarwala P. Wagner F. Chandrasekharam D. Stüben D. (2005). Impact of irrigation with As rich groundwater on soil and crops: a geochemical case study in West Bengal Delta Plain, India. *Appl. Geochem.* 20 1890–1906. 10.1016/j.apgeochem.2005.04.019

[B83] Norton G. J. Lou-Hing D. E. Meharg A. A. Price A. H. (2008). Rice–arsenate interactions in hydroponics: whole genome transcriptional analysis. *J. Exp. Bot.* 59 2267–2276. 10.1093/jxb/ern097 18453530 PMC2413274

[B84] Oancea S. Foca N. Airinei A. (2005). Effects of heavy metals on plant growth and photosynthetic activity. *Analele Univ. Al. I. Cuza.* 1 107–110.

[B85] Öncel I. Keleȿ Y. Üstün A. S. (2000). Interactive effects of temperature and heavy metal stress on the growth and some biochemical compounds in wheat seedlings. *Environ. Pollut.* 107 315–320. 10.1016/S0269-7491(99)00177-3 15092977

[B86] Ozden M. Demirel U. Kahraman A. (2009). Effects of proline on antioxidant system in leaves of grapevine (*Vitis vinifera* L.) exposed to oxidative stress by H_2_O_2_. *Sci. Hortic.* 119 163–168. 10.1016/j.scienta.2008.07.031

[B87] Pandey P. K. Yadav S. Nair S. Bhui A. (2002). Arsenic contamination of the environment: a new perspective from central-east India. *Environ. Int.* 28 235–245. 10.1016/S0160-4120(02)00022-3 12220110

[B88] Paszkowski U. Kroken S. Roux C. Briggs S. P. (2002). Rice phosphate transporters include an evolutionarily divergent gene specifically activated in arbuscular mycorrhizal symbiosis. *Proc. Natl. Acad. Sci. U.S.A.* 20 13324–13329. 10.1073/pnas.202474599 PMC130632 12271140

[B89] Phillips J. M. Hayman D. S. (1970). Improved procedures for clearing roots and staining parasitic and vesicular-arbuscular mycorrhizal fungi for rapid assessment of infection. *Trans. Br. Mycol. Soc.* 55 158–161. 10.1016/S0007-1536(70)80110-3

[B90] Principato G. B. Rosi G. Talesa V. Giovanni E. Uotila L. (1987). Purification and characterization of two forms of glyoxalase II from the liver and brain of Wistar rats. *Biochim. Biophys. Acta* 911 349–355. 10.1016/0167-4838(87)90076-8 3814608

[B91] Rahman M. M. Ng J. C. Naidu R. (2009). Chronic exposure of arsenic via drinking water and its adverse health impacts on humans. *Environ. Geochem. Health* 31 189–200. 10.1007/s10653-008-9235-0 19190988

[B92] Roychowdhury T. Uchino T. Tokunaga H. Ando M. (2002). Arsenic and other heavy metals in soils from an arsenic-affected area of West Bengal. India. *Chemosphere* 49 605–618. 10.1016/S0045-6535(02)00309-0 12430648

[B93] Ruiz-Lozano J. M. (2003). Arbuscular mycorrhizal symbiosis and alleviation of osmotic stress. New perspectives for molecular studies. *Mycorrhiza* 13 309–317. 10.1007/s00572-003-0237-6 12690537

[B94] Ruiz-Sánchez M. Aroca R. Muñoz Y. Polón R. Ruiz-Lozano J. M. (2010). The arbuscular mycorrhizal symbiosis enhances the photosynthetic efficiency and the antioxidative response of rice plants subjected to drought stress. *J. Plant Physiol.* 167 862–869. 10.1016/j.jplph.2010.01.018 20227134

[B95] Sadasivam S. Manickam A. (2008). *Biochemical Methods* 3rd Edn. New Delhi: New Age International Pvt. Limited 4–10.

[B96] Saxena M. Roy S. D. Singla-Pareek S. L. Sopory S. K. Bhalla-Sarin N. (2011). Overexpression of the glyoxalase II gene leads to enhanced salinity tolerance in *Brassica juncea*. *Open Plant Sci. J.* 5 23–28. 10.2174/1874294701105010023

[B97] Sekar I. Pal S. (2012). Rice and wheat crop productivity in the Indo-Gangetic Plains of India: changing pattern of growth and future strategies. *Indian J. Agric. Econ.* 67 238–252.

[B98] Serbinonva E. A. Packer L. (1994). Antioxidant properties of tocopherol and tocotrienol. *Methods Enzymol.* 234 354–367. 10.1016/0076-6879(94)34105-2 7808307

[B99] Shamshiri M. H. Fattahi M. (2014). Evaluation of two biochemical markers for salt stress in three pistachio rootstocks inoculated with arbuscular mycorrhiza (*Glomus mosseae*). *J. Stress Physiol. Biochem.* 10 335–346.

[B100] Sharma I. (2012). Arsenic induced oxidative stress in plants. *Biologia* 67 447–453. 10.2478/s11756-012-0024-y

[B101] Sieh D. Watanabe M. Devers E. A. Brueckner F. Hoefgen R. Krajinski F. (2013). The arbuscular mycorrhizal symbiosis influences sulfur starvation responses of *Medicago truncatula*. *New Phytol.* 197 606–616. 10.1111/nph.12034 23190168

[B102] Singh H. P. Batish D. R. Kohli R. K. Arora K. (2007). Arsenic-induced root growth inhibition in mung bean (*Phaseolus aureus* Roxb.) is due to oxidative stress resulting from enhanced lipid peroxidation. *Plant Growth Regul.* 53 65–73. 10.1007/s10725-007-9205-z

[B103] Singh N. Ma L. Q. Srivastava M. Rathinasabapathi B. (2006). Metabolic adaptations to arsenic-induced oxidative stress in *Pteris vittata* L and *Pteris ensiformis* L. *Plant Sci.* 170 274–282. 10.1016/j.plantsci.2005.08.013

[B104] Singla-Pareek S. L. Yadav S. K. Pareek A. Reddy M. K. Sopory S. K. (2008). Enhancing salt tolerance in a crop plant by overexpression of glyoxalase II. *Transgenic Res.* 17 171–180. 10.1007/s11248-007-9082-2 17387627

[B105] Smith A. H. Lingas E. O. Rahman M. (2000). Contamination of drinking-water by arsenic in Bangladesh: a public health emergency. *Bull. World Health Organ.* 78 1093–1103. 10.1590/S0042-96862000000900005 11019458 PMC2560840

[B106] Smith F. W. Mudge S. R. Rae A. L. Glassop D. (2003). Phosphate transport in plants. *Plant Soil* 248 71–83. 10.1023/A:1022376332180a

[B107] Smith I. K. Verheller T. L. Thorne C. A. (1989). Properties and functions of glutathione reductase in plants. *Physiol. Plant.* 77 449–455. 10.1111/j.1399-3054.1989.tb05666.x

[B108] Smith S. E. Christophersen H. M. Pope S. Smith F. A. (2010a). Arsenic uptake and toxicity in plants: integrating mycorrhizal influences. *Plant Soil* 327 1–21. 10.1007/s11104-009-0089-8

[B109] Smith S. E. Facelli E. Pope S. Smith F. A. (2010b). Plant performance in stressful environments: interpreting new and established knowledge of the roles of arbuscular mycorrhizas. *Plant Soil* 326 3–20. 10.1007/s11104-009-9981-5

[B110] Smith S. E. Read D. J. (2008). *Mycorrhizal Symbiosis.* San Diego, CA: Academic Press.

[B111] Smith S. E. Smith F. A. (2011). Roles of arbuscular mycorrhizas in plant nutrition and growth: new paradigms from cellular to ecosystem scales. *Annu. Rev. Plant Biol.* 62 227–250. 10.1146/annurev-arplant-042110-103846 21391813

[B112] Sobrino-Plata J. Meyssen D. Cuypers A. Escobar C. Hernández L. E. (2014). Glutathione is a key antioxidant metabolite to cope with mercury and cadmium stress. *Plant Soil* 377 369–381. 10.1007/s11104-013-2006-4

[B113] Spagnoletti F. Lavado R. S. (2015). The arbuscular mycorrhiza *Rhizophagus intraradices* reduces the negative effects of arsenic on soybean plants. *Agronomy* 5 188–199. 10.3390/agronomy5020188

[B114] Srivastava M. Ma L. Q. Singh N. Singh S. (2005). Antioxidant responses of hyper-accumulator and sensitive fern species to arsenic. *J. Exp. Bot.* 56 1335–1342. 10.1093/jxb/eri134 15781440

[B115] Srivastava S. Akkarakaran J. J. Sounderajan S. Shrivastava M. Suprasanna P. (2016). Arsenic toxicity in rice (*Oryza sativa* L.) is influenced by sulfur supply: impact on the expression of transporters and thiol metabolism. *Geoderma* 270 33–42. 10.1016/j.geoderma.2015.11.006

[B116] Srivastava S. Sharma Y. K. (2013). Impact of arsenic toxicity on black gram and its amelioration using phosphate. *ISRN Toxicol.* 2013:340925 10.1155/2013/340925 PMC3736483 23970978

[B117] Stoeva N. Bineva T. (2003). Oxidative changes and photosynthesis in oat plants grown in As-contaminated soil. *Bulg. J. Plant Physiol.* 29 87–95.

[B118] Tan S. Y. Jiang Q. Y. Zhuo F. Liu H. Wang Y. T. Li S. S. (2015). Effect of inoculation with *Glomus versiforme* on cadmium accumulation, antioxidant activities and phytochelatins of *Solanum photeinocarpum*. *PLoS ONE* 10:e0132347 10.1371/journal.pone.0132347 PMC4503595 26176959

[B119] Tao Y. Zhang S. Jian W. Yuan C. Shan X. Q. (2006). Effects of oxalate and phosphate on the release of arsenic from contaminated soils and arsenic accumulation in wheat. *Chemosphere* 65 1281–1287. 10.1016/j.chemosphere.2006.04.039 16750554

[B120] Tong J. Guo H. Wei C. (2014). Arsenic contamination of the soil–wheat system irrigated with high arsenic groundwater in the Hetao Basin, Inner Mongolia, China. *Sci. Total Environ.* 496 479–487. 10.1016/j.scitotenv.2014.07.073 25108250

[B121] Trotta A. Falaschi P. Cornara L. Minganti V. Fusconi A. Drava G. (2006). Arbuscular mycorrhizae increase the arsenic translocation factor in the As hyperaccumulating fern *Pteris vittata* L. *Chemosphere* 65 74–81. 10.1016/j.chemosphere.2006.02.048 16603227

[B122] Ultra V. U. Tanaka S. Sakurai K. Iwasaki K. (2007). Effects of arbuscular mycorrhiza and phosphorus application on arsenic toxicity in sunflower (*Helianthus annuus* L.) and on the transformation of arsenic in the rhizosphere. *Plant Soil* 29 29–41. 10.1007/s11104-006-9087-2

[B123] Van Assche F. Clijsters H. (1990). Effects of metals on enzyme activity in plants. *Plant Cell Environ.* 13 195–206. 10.1111/j.1365-3040.1990.tb01304.x

[B124] Velikova V. Yordanov I. Edreva A. (2000). Oxidative stress and some antioxidant systems in acid rain-treated bean plants: protective role of exogenous polyamines. *Plant Sci.* 151 59–66. 10.1016/S0168-9452(99)00197-1

[B125] Verbruggen N. Hermans C. (2008). Proline accumulation in plants: a review. *Amino Acids* 35 753–759. 10.1007/s00726-008-0061-6 18379856

[B126] Wang S. Y. Jiao H. (2000). Scavenging capacity of berry crops on superoxide radicals, hydrogen peroxide, hydroxyl radicals, and singlet oxygen. *J. Agric. Food Chem.* 48 5677–5684. 10.1021/jf000766i 11087538

[B127] Wu Q. S. Zou Y. N. Xia R. X. (2006). Effects of water stress and arbuscular mycorrhizal fungi on reactive oxygen metabolism and antioxidant production by citrus (*Citrus tangerine*) roots. *Eur. J. Soil Biol.* 42 166–172. 10.1016/j.ejsobi.2005.12.006

[B128] Xia Y. S. Chen B. D. Christie P. Wang Y. S. Li X. L. (2007). Arsenic uptake by arbuscular mycorrhizal maize (*Zea mays* L.) grown in an arsenic-contaminated soil with added phosphorus. *J. Environ. Sci.* 19 1245–1251. 10.1016/S1001-0742(07)60203-4 18062425

[B129] Yang Y. Han X. Liang Y. Ghosh A. Chen J. Tang M. (2015). The combined effects of arbuscular mycorrhizal fungi (AMF) and lead (Pb) stress on Pb accumulation, plant growth parameters, photosynthesis, and antioxidant enzymes in *Robinia pseudoacacia* L. *PLoS ONE* 10:e0145726 10.1371/journal.pone.0145726 PMC4689355 26698576

[B130] Yu Y. Zhang S. Huang H. Luo L. Wen B. (2009). Arsenic accumulation and speciation in maize as affected by inoculation with arbuscular mycorrhizal fungus *Glomus mosseae*. *J. Agric. Food Chem.* 57 3695–3701. 10.1021/jf900107y 19296577

[B131] Zhang W. Lin K. Zhou J. Zhang W. Liu L. Zhang Q. (2014). Cadmium accumulation, sub-cellular distribution and chemical forms in rice seedling in the presence of sulfur. *Environ. Toxicol. Pharmacol.* 37 348–353. 10.1016/j.etap.2013.12.006 24388908

[B132] Zhang X. Ren B. H. Wu S. L. Sun Y. Q. Lin G. Chen B. D. (2015). Arbuscular mycorrhizal symbiosis influences arsenic accumulation and speciation in *Medicago truncatula* L. in arsenic-contaminated soil. *Chemosphere* 119 224–230. 10.1016/j.chemosphere.2014.06.042 25016555

[B133] Zhao F. J. McGrath S. P. Meharg A. A. (2010). Arsenic as a food chain contaminant: mechanisms of plant uptake and metabolism and mitigation strategies. *Ann. Rev. Plant Biol.* 61 535–559. 10.1146/annurev-arplant-042809-112152 20192735

[B134] Zhu Y. G. Sun G. X. Lei M. Teng M. Liu Y. X. Chen N. C. (2008). High percentage inorganic arsenic content of mining impacted and nonimpacted Chinese rice. *Environ. Sci. Technol.* 42 5008–5013. 10.1021/es8001103 18678041

[B135] Zou Y. N. Huang Y. M. Wu Q. S. He X. H. (2015). Mycorrhiza-induced lower oxidative burst is related with higher antioxidant enzyme activities, net H_2_O_2_ effluxes, and Ca^2+^ influxes in trifoliate orange roots under drought stress. *Mycorrhiza* 25 143–152. 10.1007/s00572-014-0598-z 25085218

