# Supplementary figures and images for: Arbuscular Mycorrhiza Augments Arsenic Tolerance in Wheat (Triticum aestivum L.) by Strengthening Antioxidant Defense System and Thiol Metabolism
Source: Front Plant Sci. 2017 Jun 8;8:906. doi: 10.3389/fpls.2017.00906 (PMC5462957; doi:10.3389/fpls.2017.00906)

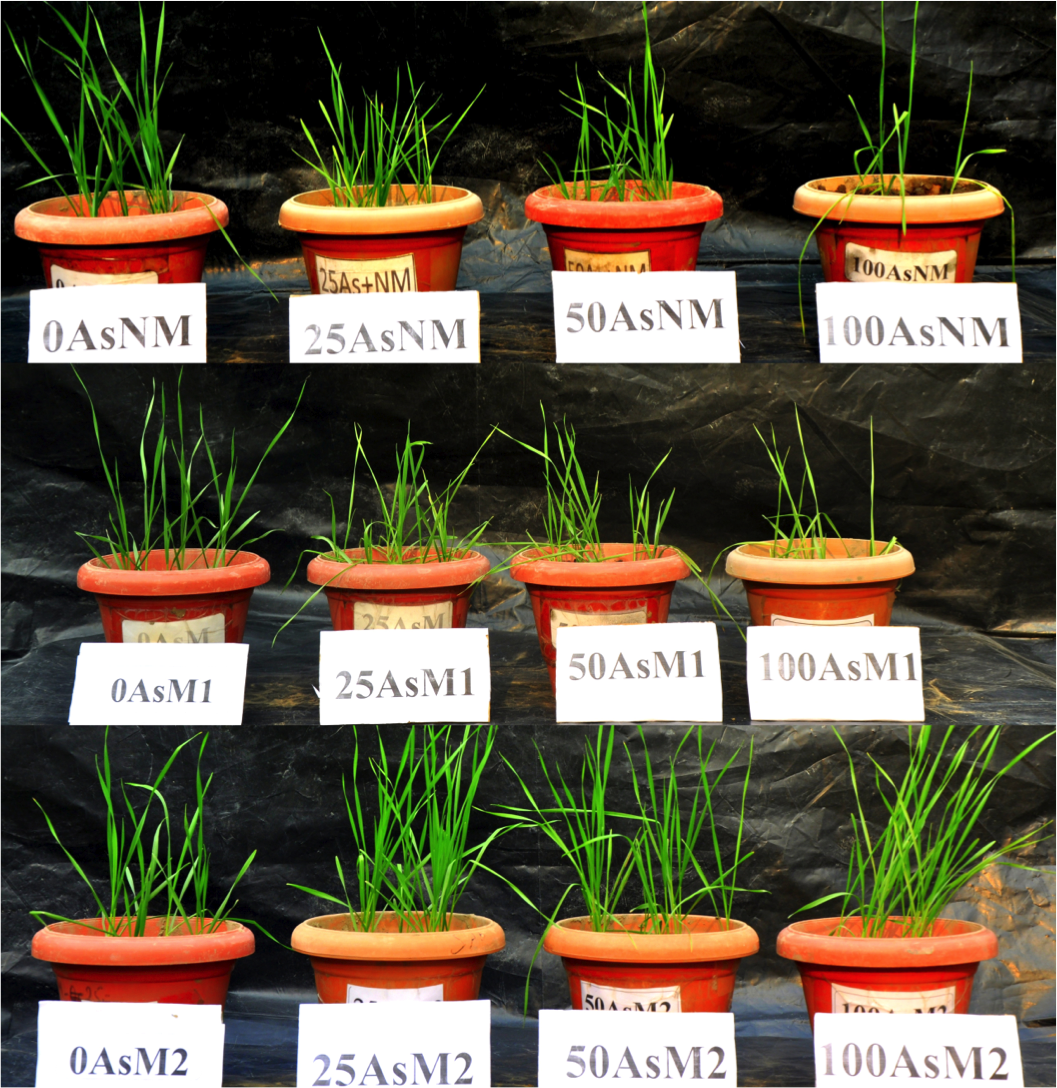

Supplement: FIGURE S1 — Pictorial representation of various treatments of As and AMF innoculation in Triticum aestivum L. var. HD-2967. [file Image_1.TIFF]

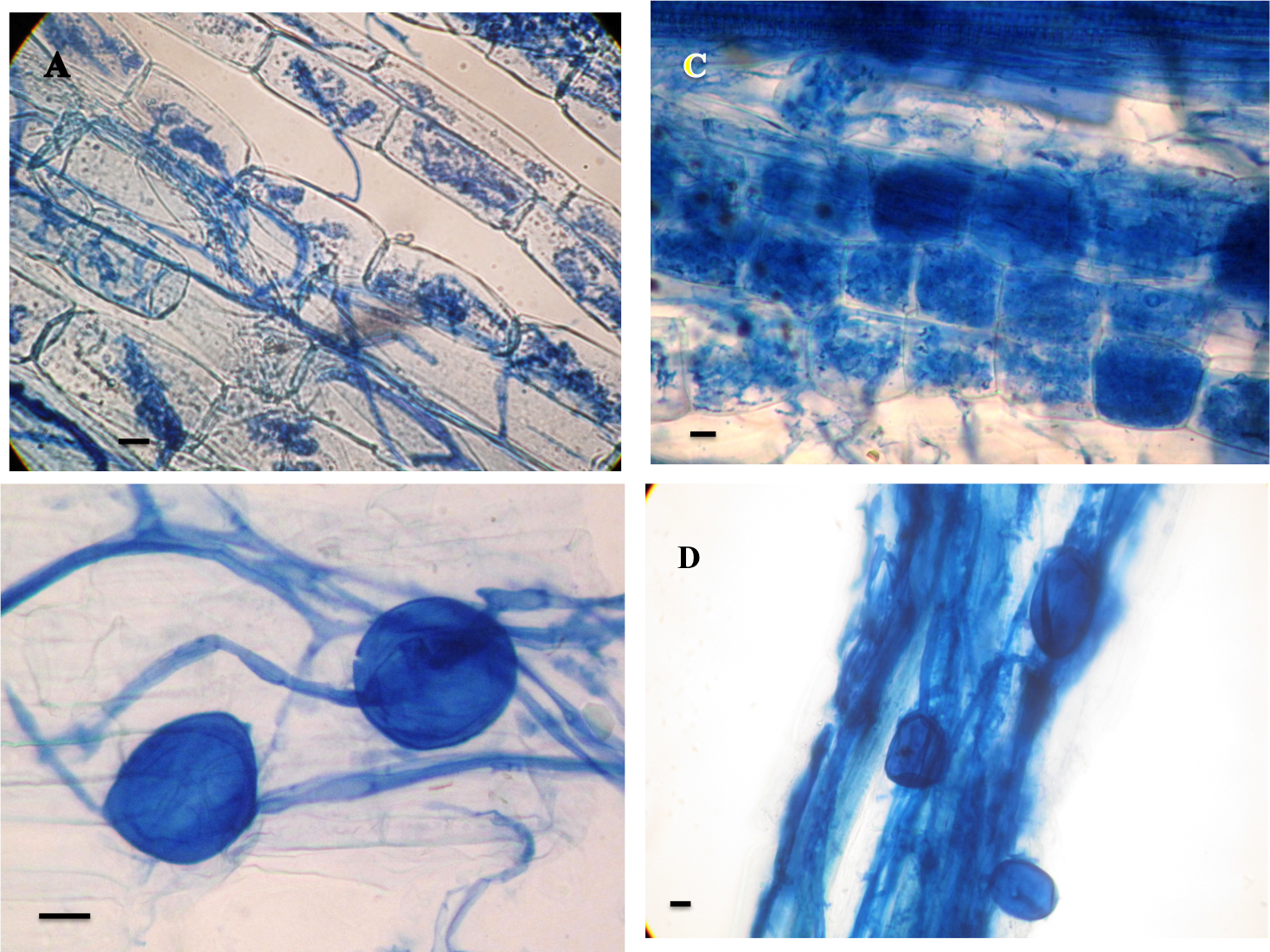

Supplement: FIGURE S2 — Histochemical staining showing AMF colonization in root cortical cells of Triticum aestivum L. var. HD-2967 with (A) arbuscules and (B) vesicles of Rhizoglomus intraradices; (C) arbuscules and (D) vesicles of Glomus etunicatum stained with trypan blue. Bar = 50 μm. [file Image_2.TIFF]
